# Non-targeted GC–MS metabolomics-based differences in Indica rice seeds of different varieties

**DOI:** 10.1186/s12870-024-05255-6

**Published:** 2024-06-08

**Authors:** Dahu Zhou, Hao Jing, Jun Yuan, Mingming Zhou, Lin Liu, Junru Fu, Linjuan Ouyang, Jie Xu, Jianmin Bian, Haihui Fu, Haohua He

**Affiliations:** 1 Key Laboratory of Crop Physiology, Ecology and Genetic Breeding, Ministry of Education, College of Agronomy, Jiangxi Agricultural University, Nanchang, 330045 China; 2 School of Nursing, Jiangxi University of Chinese Medicine, Nanchang, 330004 China

**Keywords:** Rice seeds, Different varieties, Non-targeted GC–MS, Amino acids, Sugars

## Abstract

Rice seeds of different varieties exhibited distinct metabolic profiles in our study. We analyzed the metabolites in seeds of six rice varieties (CH, HM, NX, YX, HY, and MX) using non-targeted GC–MS. Our findings revealed that amino acids, sugars, and organic acids were predominant in all varieties, with significant differences observed in CH compared to the others. Specifically phenylalanine and glycine content differed notably in NX and YX, respectively. Additionally, 1,5-anhydroglucitol content in NX, and glutamate, aspartate, and lactulose in NX, YX, HM, HY, and MX were up-regulated. Due to the biological functions of these amino acids and sugars, these indicated that compared to CH, rice of NX were more conducive to metabolism of carbohydrate and fat, and healthy growth maintenance in the human body, but mightThese variations suggest that NX rice may be more beneficial for carbohydrate and fat metabolism and overall health maintenance compared to CH. However, it may not be suitable for diabetic patients. YX rice may not be an ideal glycine supplement, rice ofwhile HM, HY, and MX rice could serve as potential lactulose sources. Furthermore, NX and YX rice exhibited higher levels of main storage proteins compared to CH. This study offers valuable insights into the metabolic differences among various rice varieties.

**Supplementary Information:**

The online version contains supplementary material available at 10.1186/s12870-024-05255-6.

## Introduction

Metabolomics is characterized by its short analysis time, high accuracy, and wide coverage, allowing for the comprehensive detection of small molecular metabolites in samples and the effective integration of sample information [1]. Non-targeted metabolomics, which can detect a variety of metabolites, provides broad insights into metabolite profiles in samples and has been widely applied in various disciplines such as medical, plant, and microbiology [2], For example, Huang used GC–MS to study the effect of CCl4 on liver injury [3], and Qian used GC–MS to determine the amino acid content in human plasma [4].In the context of rice, numerous studies have utilized non-targeted metabolomics to investigate the metabolic response to abiotic and biotic stress, as well as growth and development [5–7]. These studies serve as the foundation for further research on metabolomics-based comparisons in rice seeds of different varieties.

Rice, as one of main the grain crops, has been cultivated in China for over 7000 years. Jiangxi province, a major agricultural region, predominantly grows rice, which holds high economic value. Rice cultivation covers more than 90% of the total cultivated area of grain crops in Jiangxi, with rice yield contributing over 95% of the total grain crop yield [8].The “early Indica and late Japonica” cultivation pattern of double cropping rice has led to significant advancements in breeding efforts in Jiangxi province [9]. By 2020, 11 rice varieties in Jiangxi were designated as "super rice" and high-quality rice varieties received gold medals for taste quality evaluations [8]. Understanding the differences in variety-related characteristic components is crucial for optimizing the development and utilization of novel rice cultivars [10, 11].

Metabolites in rice seeds not only reflect the overall metabolic state, but also significantly impact the quality of rice [12, 13]. However, the metabolites in rice seeds of different varieties have not been extensively studied. In this study, an untargeted metabolomics approach using gas chromatography-mass spectrometry (GC–MS) was employed to differentiate six Indica rice varieties originating from Jiangxi Province. The objectives of this study are: 1) to present the metabolic profiles of the six Indica rice varieties; and 2) to elucidate the metabolomics-based differences among the six Indica rice varieties. This study aims to provide new insights into the differences of rice varieties, and serve as a valuable reference for the exploration and breeding of high-quality rice germplasm resources.

## Materials and methods

### Rice materials

Rice seeds of Changhui 871, Huangxiang Yujing, Nongxiang 39, Daoxiang, Huangxiang Yousi, and Meixiangzhan 2 were sown in the agricultural technological garden of Jiangxi Agricultural University with unified water and fertilizer application on June 18, 2022. Samples of Changhui 871 (CH), Huaxiang Madi (HM), Nongxiang 39 (NX), Yahe Xiang (YX), Huaxiang Yousi (HY), and Meixiangzhan 2 (MX) were collected sequentially based on the maturity of each variety.

### GC–MS analysis

The metabolites were extracted and identified with GC–MS according to our previously reported methods [14]. A total of 50 mg brown rice was introduced into a 2 mL centrifuge tube. Subsequently, 0.5 mL of methanol-chloroform (3:1, v/v) and 10 μL of ribitol (2 mg mL^−1^ stock in water, internal quantitative standard) were introduced into the tube. The mixture was vortexed for 30 s, ground at 45 HZ for 4 min, and kept in an ice bath for 5 min. Repeat these three steps for three times. Then the mixture was centrifuged at 12,000 rpm for 15 min, with 300 μL of the polar phase sample collected independently into 1.5 mL centrifugal tube and dried in a benchtop centrifugal concentrator (LNG-T98, Huamei Biochemical Instrument Factory, Taicang City, China) for 3.0 h until thoroughly dried. Methoximation (incubating the dried fraction at 80 °C for 30 min with a 60 μL of 20 mg mL^−1^ methoxyamine hydrochloride) and trimethylsilylation (incubating the dried fraction at 70 °C for 1.5 h with a 70 μL BSTFA (BSTFA: TMCS = 99:1, v/v)) of the upper polar phase were carried out, in turn. Cooling the solution down to room temperature, 5 μL FAMEs were added. An 80 μL supernatant of all the samples mixed into the QC sample. The GC–MS analysis was carried out according to Yuan et al. [15, 16].

Sample derivatization was conducted with Gas Chromatography Time-Of-Flight Mass Spectrometry (GC-TOF–MS, an Agilent 7890 gas chromatograph system coupled with a Pegasus HT time-of-flight mass spectrometerin Novogene Co., Ltd. (Beijing, China)) with a DB-5MS capillary column (30 m × 0.25 mm × 250 mm) (Agilent JW Scientific, Folsom, CA)). A 1 μL aliquot of each sample was injected into the DB-5MS capillary column. The GC oven temperature was adjusted to 50 °C; after injection for 1 min, the temperature of oven was raised from 310 °C at 10 °C min^−1^ for 8 min. The injector and ion source temperatures were adjusted to 280 °C and 250 °C, respectively. Helium was applied as the carrier gas at a constant rate of 3.0 mL min^−1^. Measurements were achieved with an electron impact at -70 eV in full-scan mode with a mass scan range of 50–500 m z^−1^.

Raw data analysis, including peak extraction, baseline adjustment, deconvolution, alignment and integration, was conducted with Chroma TOF (V 4.3x, LECO) software. LECO-Fiehn Rtx5 database was used for metabolite identification by matching the mass spectrum and retention index. And the peaks detected in less than half of QC samples or RSD > 30% in QC samples was removed.

### Data analysis

Analysis of variance (ANOVA) was carried out to evaluate the differences of metabolites levels between CH and other groups. Unsupervised principal component analysis (PCA), and supervised partial least squares discrimination analysis (PLS-DA) were performed to assess the effects of different varieties on the metabolic data. ANOVA of the cross validated residuals (CV-ANOVA) or permutation tests (200) was conducted to validate the PLS-DA model. A R2 value > Q2 value, and the intercept between the Q2 regression line and the Y-axis is less than 0 denoted that the models were highly significant. Furthermore, the variable importance in projection (VIP) was critical for explaining the data, and obtained with PLS-DA. Metabolites, with a VIP of above 1.0 and a *p* value of below 0.05, were selected as discriminating metabolites, which played important roles in distinguishing rice of different varieties. According to the results of the pathway analysis involving the discriminating metabolites, the potential metabolic target pathways, with the value of pathway impact value (PI) > 0.1, were filtered out from the pathway topology.

AMOVA was performed by SPSS 20.0 (SPSS Inc., USA). PCA and PLS-DA were performed with SIMCA-P version 14.1 (Umetrics, Sweden), and the pathway analysis involving discriminating metabolites was carried out with the free online software MetaboAnalyst 4.0 (http://www.metaboanalyst. ca/faces/ModuleView.xhtml). And the metabolic map of discriminating metabolites involved in potemtial targeted pathways was charted. All data were log10-transformed to improve normality prior to analysis.

## Results

### Metabolic profiles in rice seeds of different varieties

**Fig. 1:**
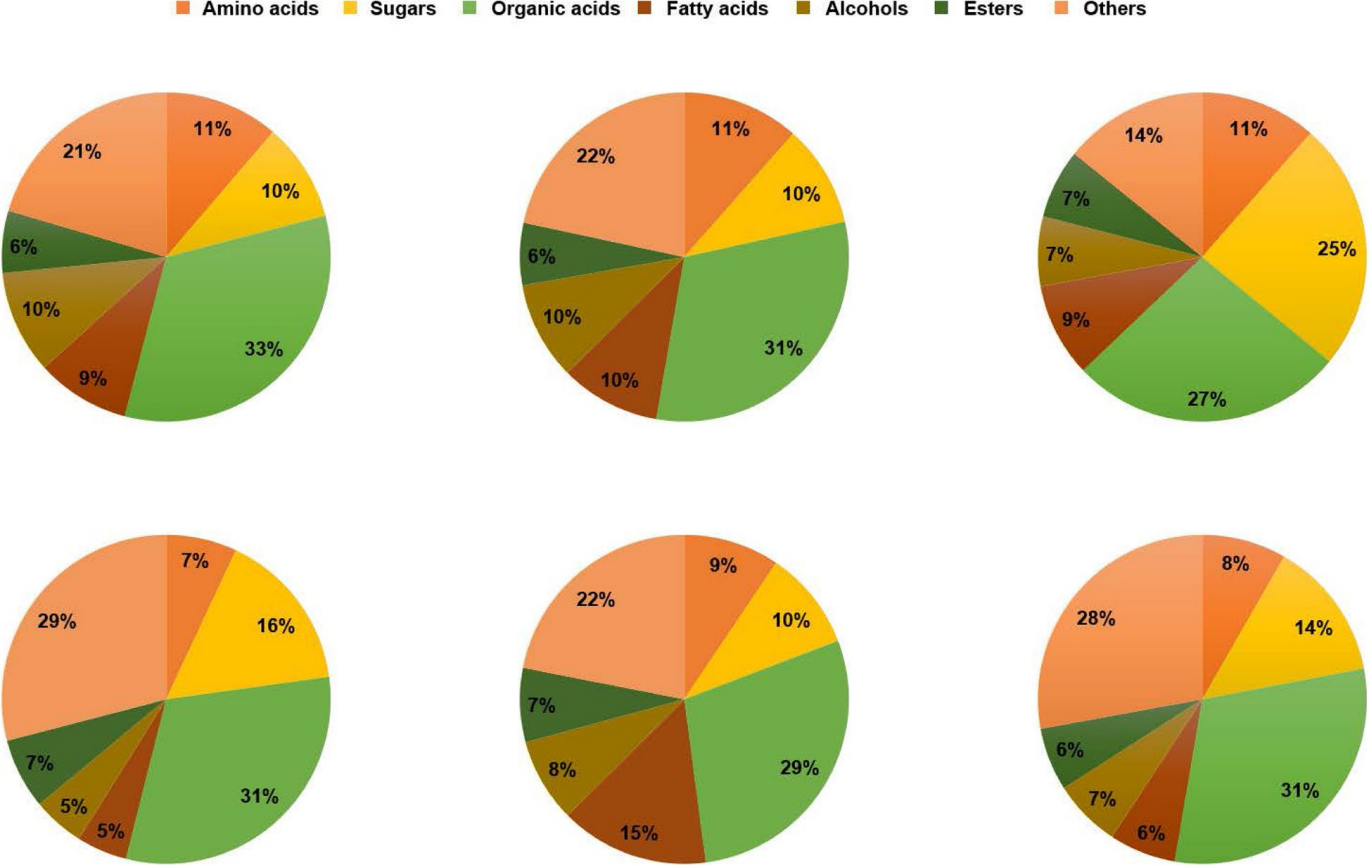
Percentages of different metabolites in rice seeds of different types. A, B, C, D, E, and F stand for percentages of different metabolites in rice seeds of CH, HM, NX, YX, HY and MX, respectively, and CH, HM, NX, YX, HY and MX stand for Changhui 871, Huaxiang Madi, Nongxiang 39, Yahe xiang, Huaxiang Yousi, and Meixiangzhan 2, respectively, the same below

All the Pearson correlation coefficient of the QC samples were above 0.9 (Figure S1). A total of 221 metabolites were detected in the study, which were classified into amino acids (7%-11%), sugars (10%-25%), organic acids (27%-33%), fatty acids (5%-15%), alcohols (5%-10%), esters(6%-7%) and others (14%-29%). Among them, organic acids (27%-33%), amino acids (7%-11%), and sugars (10%-25%) accounted for the most in rice seeds of different varieties (Fig. 1; Table S1).

**Fig. 2:**
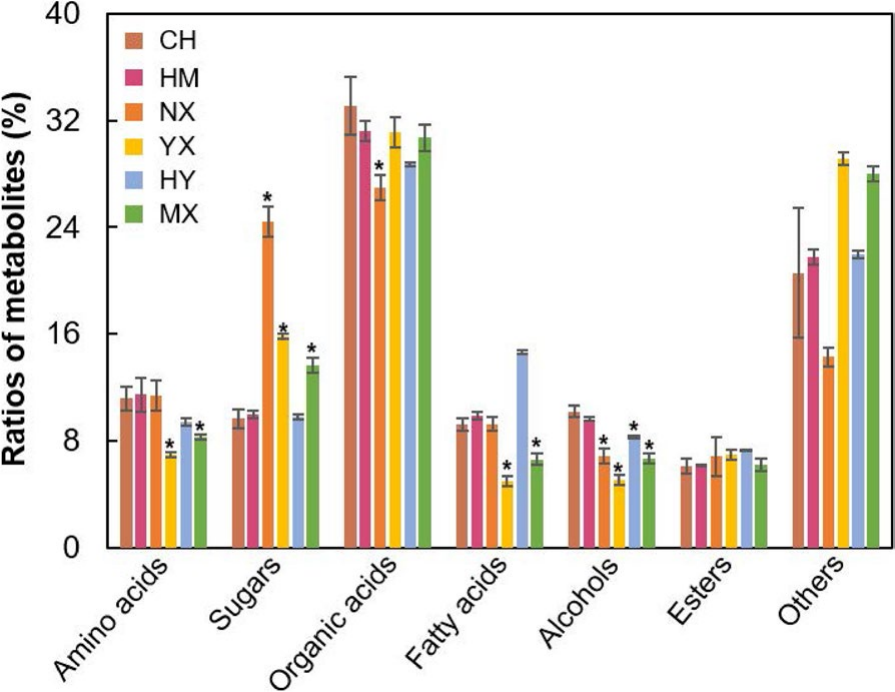
Percentages of different kinds of metabolites in rice seeds of different types. CH, HM, NX, YX, HY and MX stand for Changhui 871, Huaxiang Madi, Nongxiang 39, Yahe xiang, Huaxiang Yousi, and Meixiangzhan 2, respectively, the same below; * stand for significant differences in percentages between CH and others

There were no significant differences in percentages of the seven kinds of metabolites between CH and the other varieties. However, compared to CH, the percentage of sugars was higher, and fatty acids and alcohols were lower in NX; the percentages of amino acids and sugars were higher, and fatty acids and alcohols were lower in YX; the percentage of fatty acids was higher, and alcohols was lower in HY; and the percentages of amino acids, fatty acids and alcohols were lower, while the percentage of sugars was higher in MX (*p* < 0.05) (Fig. 2).

### Discriminating metabolites in comparison with CH

**Fig. 3:**
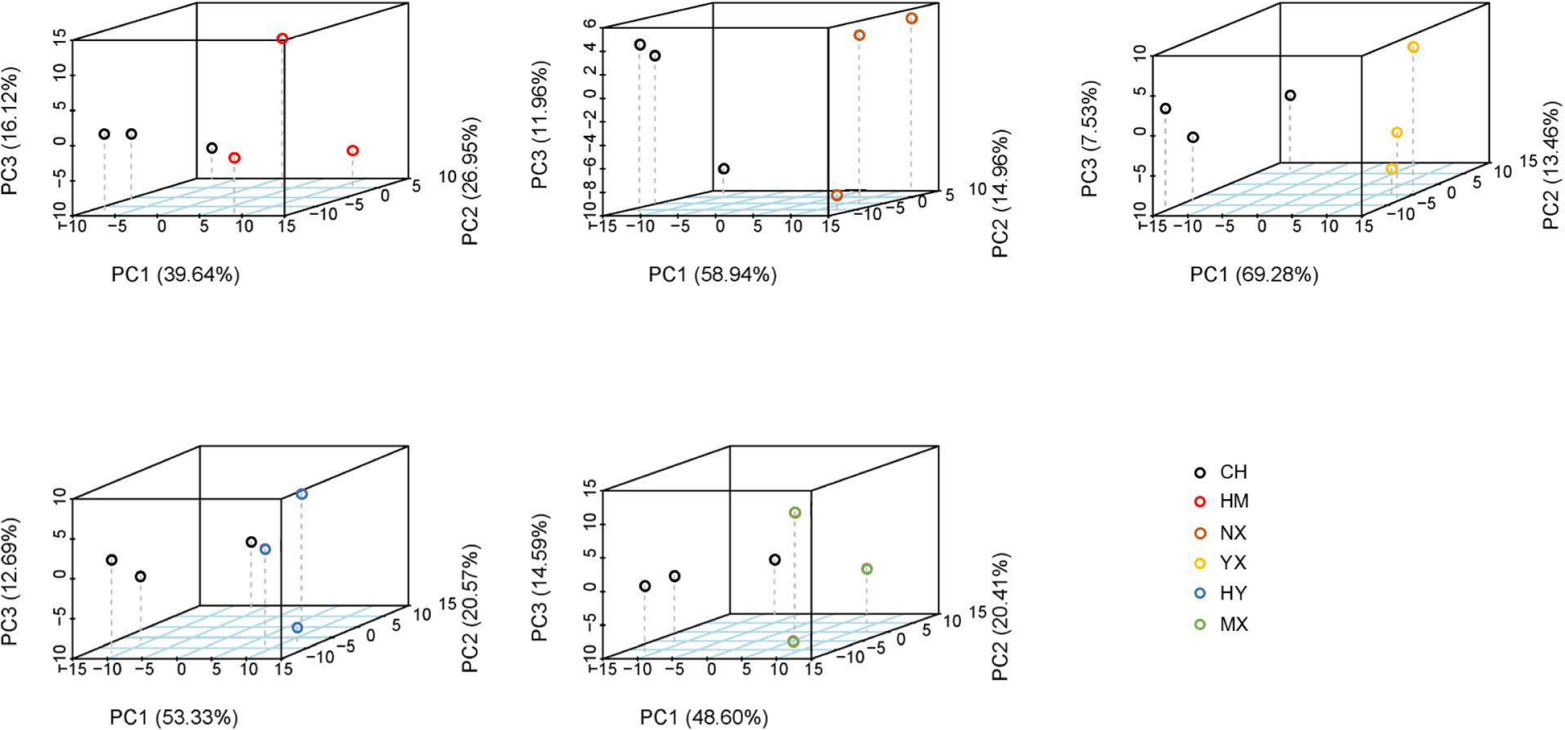
PCA plots of metabolite data in rice seeds between CH and other types. A, B, C, D, and E stand for PCA plots of metabolite data in rice seeds between CH and HM, NX, YX, HM, MX, respectively

**Fig. 4:**
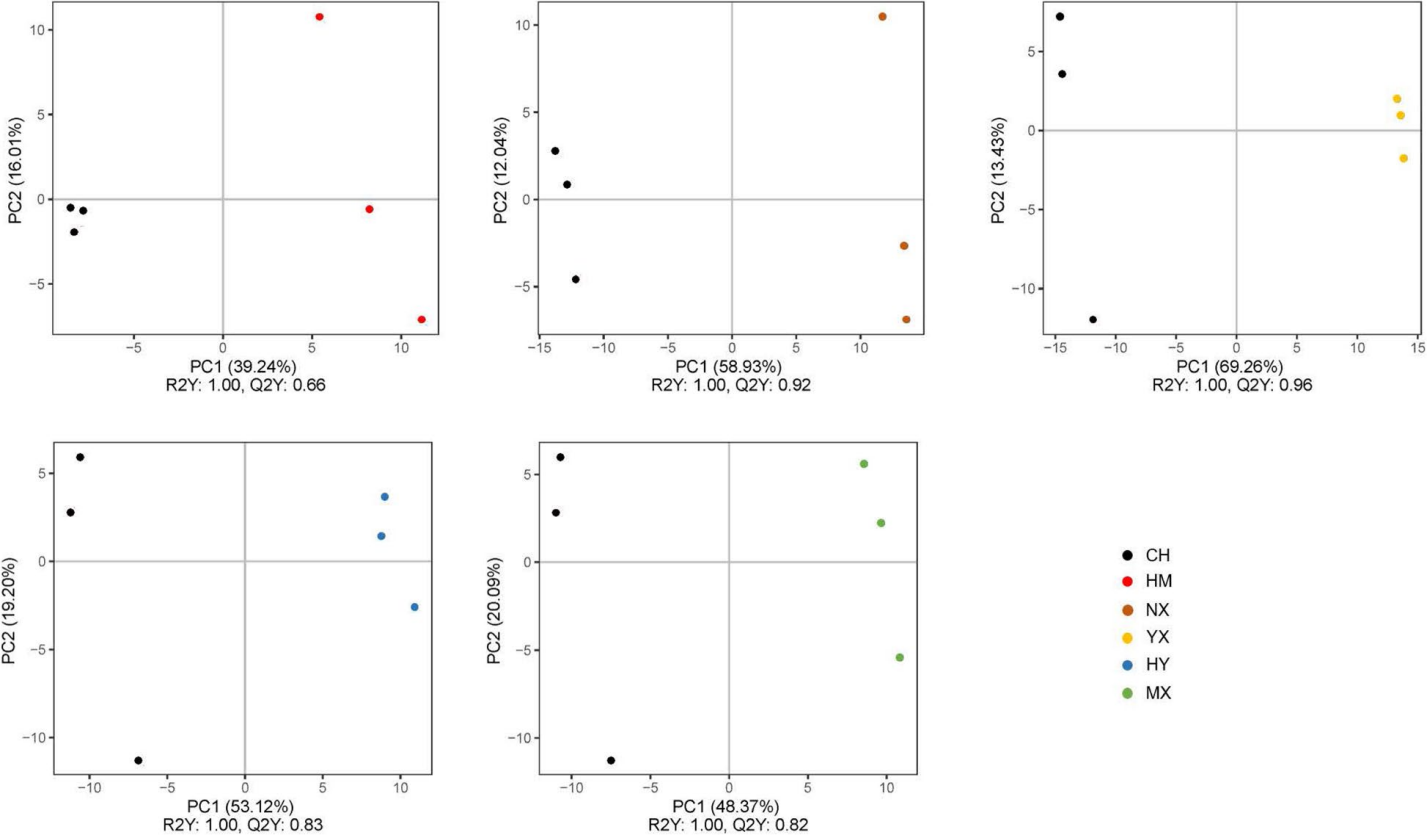
PLS-DA plots of metabolite data in rice seeds between CH and other types. A, B, C, D, and E stand for PLS-DA plots of metabolite data in rice seeds between CH and HM, NX, YX, HM, MX, respectively

**Fig. 5:**
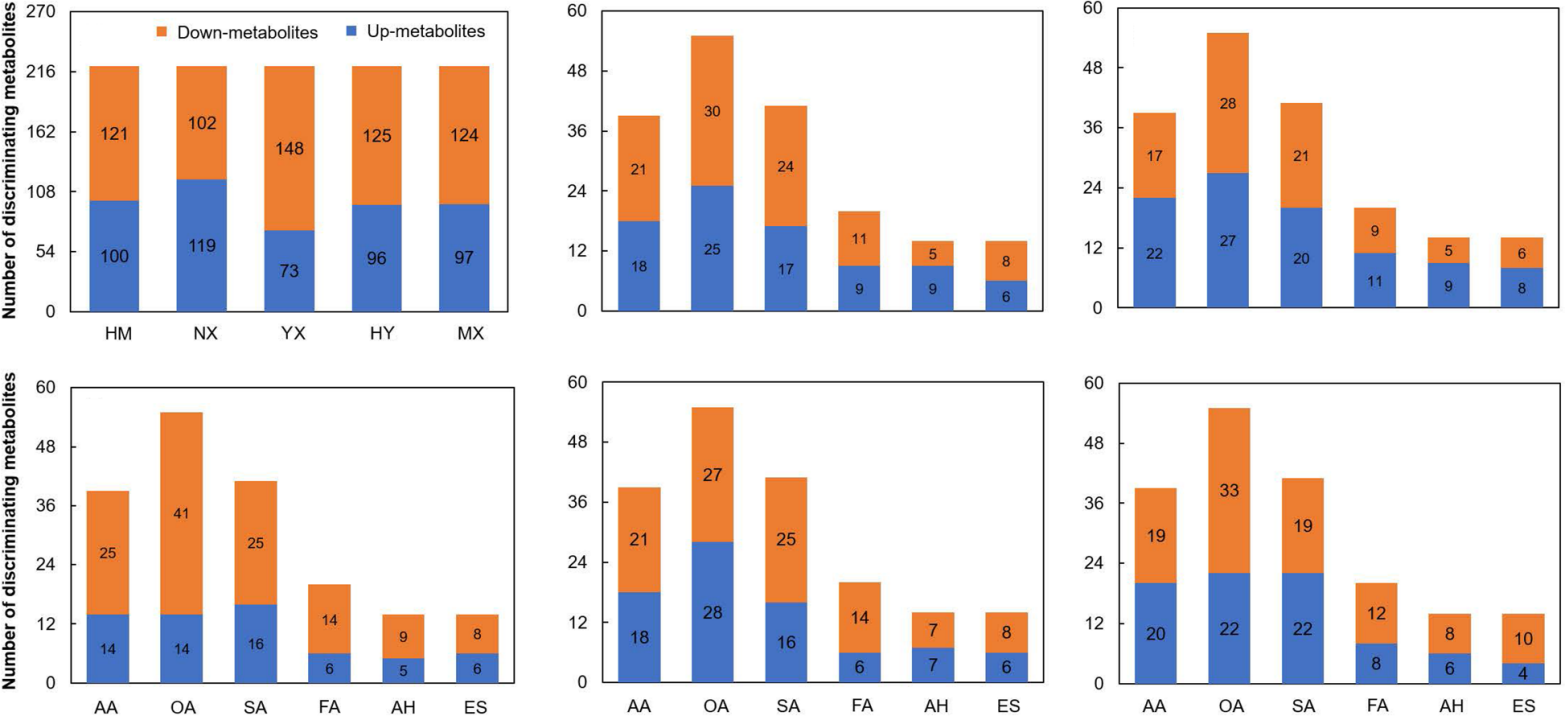
The number of down- and up- metabolites in discriminating metabolites of rice seeds between CH and other types. A stand for all the down- and up- metabolites in discriminating metabolites of rice seeds between CH and others; B, C, D, E and F stand for down- and up- metabolites in discriminating metabolites in HM, NX, YX, HY, and MX, respectively, compared to CH; AA, OA, SA, FA, AH, and ES stand for amino acids, organic acids, sugars, fatty acids, alcohols and esters, respectively

Based on the results of PCA and significant PLS-DA (R^2^ > 0.7 and Q^2^ > 0.5), rice seed samples in CH and the other varieties (HM, NX, YX, HY, and MX) could be significantly separated (Figs. 3, 4 and S2). With VIP > 1 and *p* < 0.05, compared to CH, 18, 42, 45, 30, and 28 discriminating metabolites (DMs) were detected in HM, NX, YX, HY, and MX, respectively (Fig. 5, Table S2), with varying numbers of up and down DMs between different groups. Most of the DMs were up regulated in NX, while most were down regulated in YX (Fig. 5).

**Fig. 6:**
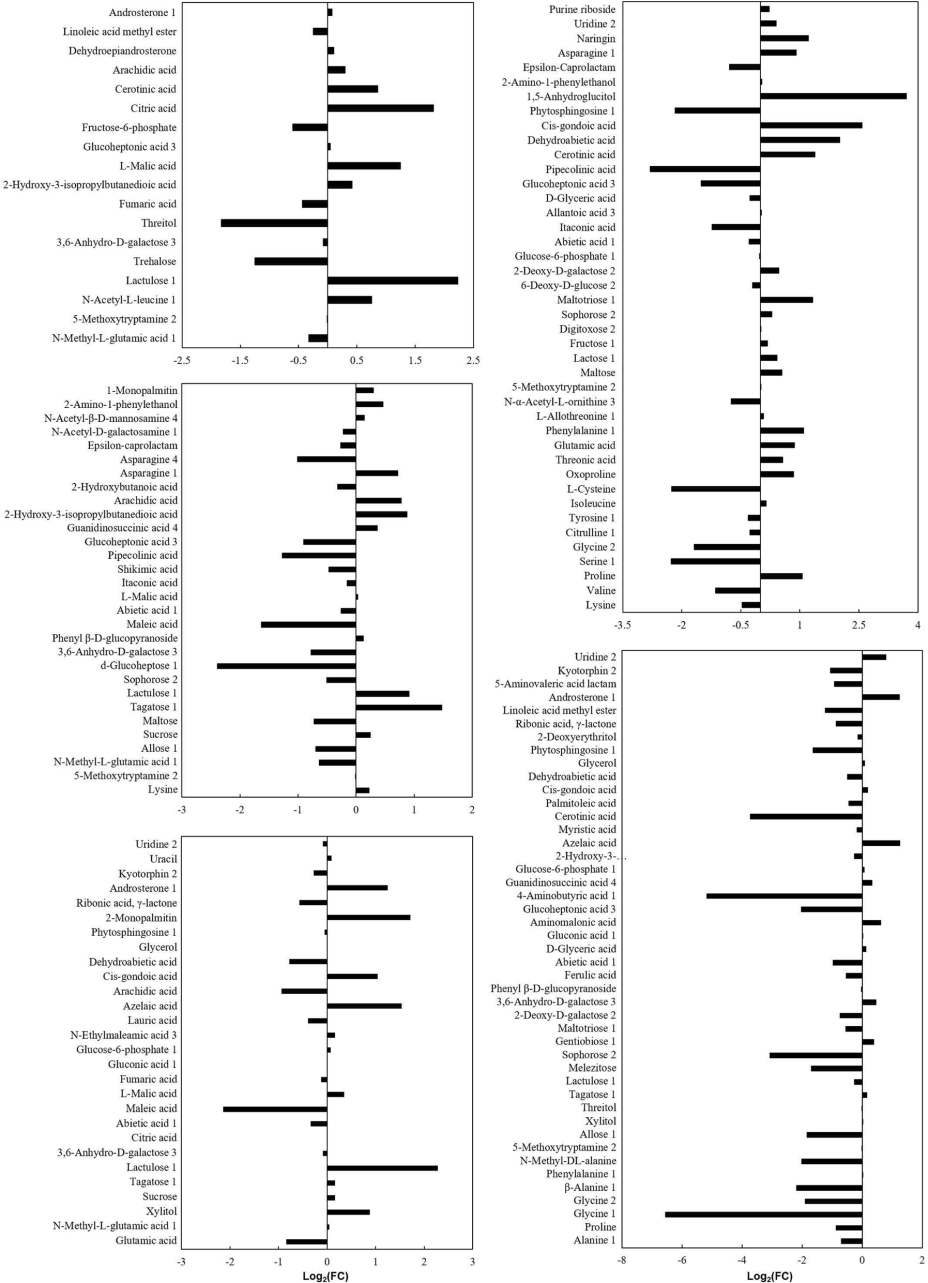
Discriminating metabolites in rice seeds between CH and other types. A, B, C, D and E stand for discriminating metabolites in rice seeds of HM, NX, YX, HY, and MX, respectively, compared to CH. Log_2_(FC), Log_2_ transform of metabolite concentration in rice seeds between other types and CH

Amino acids, organic acids, and sugars were the predominant metabolites, contributing more to the separation between CH and the other varieties (Fig. 6). The predominant up-regulated amino acids and amino acid derivatives were N-acetyl-L-leucine in HM (FC = 1.69), phenylalanine in NX (FC = 2.13), asparagine in HY (FC = 1.65), and N-methyl-L-glutamic acid in MX (FC = 1.03). The predominant up-regulated sugars were lactulose in HM (FC = 4.69) and MX (FC = 4.87), 1,5-anhydroglucitol in NX (FC = 13.02), and tagatose in HY (FC = 2.78). The predominant up-regulated organic acids were citric acid in HM (FC = 3.51), dehydroabietic acid in NX (FC = 4.02), arachidic acid in HY (FC = 1.71), and 2-monopalmitin in MX (FC = 3.29) (Fig. 5; Table S2). And in YX, the top three down-regulated DMs were glycine (FC = 0.01), 4-aminobutyric acid (FC = 0.03), and cerotinic acid (FC = 0.08) (Fig. 5; Table S2).

### Metabolic pathways involving all discriminating metabolites

To identify most important metabolic pathways, pathway analysis of DMs between CH and other varieties were carried out with the Kyoto Encyclopedia of Genes and Genomes (KEGG) database. The results showed that 14 potential target metabolic pathways with PI > 0.1 were determined (Table 1). Most of them were amino acid metabolisms, including arginine biosynthesis, arginine and proline metabolism, glutathione metabolism, β-alanine metabolism, phenylalanine metabolism, glycine, serine and threonine metabolism, alanine, aspartate and glutamate metabolism, and tyrosine metabolism (Table 1). And some second metabolisms were also involved (e.g., isoquinoline alkaloid biosynthesis, butanoate metabolism, glyoxylate and dicarboxylate metabolism) as well as starch and sucrose metabolism.

**Table 1:** Results of pathway analysis involving all discriminating metabolites in rice seeds of different varieties

Group	Pathway	Total Cmpd	Hits	Raw p	Holm adjust	FDR	Impact
HM vs. CH	Tyrosine metabolism	16	1	0.13	1.00	1.00	0.11
	Pyruvate metabolism	22	2	0.01	1.00	0.36	0.15
	Citrate cycle (TCA cycle)	20	3	0.00	0.05	0.05	0.18
NX vs. CH	Isoquinoline alkaloid biosynthesis	6	1	0.14	1.00	1.00	0.50
	Phenylalanine metabolism	11	1	0.24	1.00	1.00	0.47
	Alanine, aspartate and glutamate metabolism	22	2	0.10	1.00	0.84	0.32
	Glycine, serine and threonine metabolism	33	3	0.04	1.00	0.84	0.23
	Glyoxylate and dicarboxylate metabolism	29	3	0.03	1.00	0.75	0.20
	Arginine biosynthesis	18	2	0.07	1.00	0.84	0.17
	Arginine and proline metabolism	34	2	0.20	1.00	1.00	0.14
	Glutathione metabolism	26	4	0.00	0.28	0.14	0.14
	Starch and sucrose metabolism	22	1	0.42	1.00	1.00	0.14
	Tyrosine metabolism	16	1	0.33	1.00	1.00	0.11
YX vs. CH	Phenylalanine metabolism	11	1	0.21	1.00	1.00	0.47
	β-Alanine metabolism	18	1	0.33	1.00	1.00	0.25
	Glycine, serine and threonine metabolism	33	2	0.16	1.00	1.00	0.23
	Glyoxylate and dicarboxylate metabolism	29	2	0.13	1.00	1.00	0.15
	Butanoate metabolism	17	1	0.31	1.00	1.00	0.14
	Starch and sucrose metabolism	22	1	0.38	1.00	1.00	0.14
	Alanine, aspartate and glutamate metabolism	22	2	0.08	1.00	1.00	0.13
HY vs. CH	Pyruvate metabolism	22	1	0.27	1.00	1.00	0.15
MX vs. CH	Alanine, aspartate and glutamate metabolism	22	2	0.05	1.00	0.76	0.33
	Starch and sucrose metabolism	22	2	0.05	1.00	0.76	0.23
	Citrate cycle (TCA cycle)	20	3	0.00	0.34	0.34	0.18
	Pyruvate metabolism	22	2	0.05	1.00	0.76	0.15
	Glyoxylate and dicarboxylate metabolism	29	3	0.01	0.98	0.50	0.12
	Tyrosine metabolism	16	1	0.23	1.00	1.00	0.11

CH, HM, NX, YX, HY and MX stand for Changhui 871, Huaxiang madi, Nongxiang 39, Yahe xiang, Huaxiang Yousi, and Meixiangzhan 2, respectively; All pathways shown in the table are potential target metabolic pathways with pathway impacts (PI) of above 0.1; Total Cmpd, total number of compounds in the pathway; Hits, the number of actually matched compound in the pathway; Holm adjust, *p* value adjusted by Holm-Bonferroni method; FDR, *p* value adjusted using False Discovery Rate; Impact, pathway impact value

Besides, potential target metabolic pathways in rice seeds of different varieties were different, and most of them were amino acid metabolisms, sugar metabolisms, and organic acid metabolisms. There were three potential target metabolic pathways in HM, e.g., tyrosine metabolism, pyruvate metabolism, and citrate cycle (TCA cycle); ten potential target metabolic pathways in NX, e.g., glutathione metabolism, glycine, serine and threonine metabolism, glyoxylate and dicarboxylate metabolism; seven potential target metabolic pathways in YX, e.g., glycine, serine and threonine metabolism, glyoxylate and dicarboxylate metabolism, and alanine, aspartate and glutamate metabolism; one potential target metabolic pathway (pyruvate metabolism) in HY; six potential target metabolic pathways in MX, including citrate cycle (TCA cycle), glyoxylate and dicarboxylate metabolism, and Pyruvate metabolism (Table 1). Additionally, a metabolic map was developed based on these results (Fig. 6).

## Discussion

Rice seeds of different varieties hold different metabolic profiles [17]. In the study, we would reveal the differences among the six varieties via comparing the predominant metabolites (e.g., amino acids, sugars, and organic acids), especially the predominant DMs, and the main targeted metabolic pathways.

### Metabolome-based global responses in rice seeds of different varieties

**Fig. 7:**
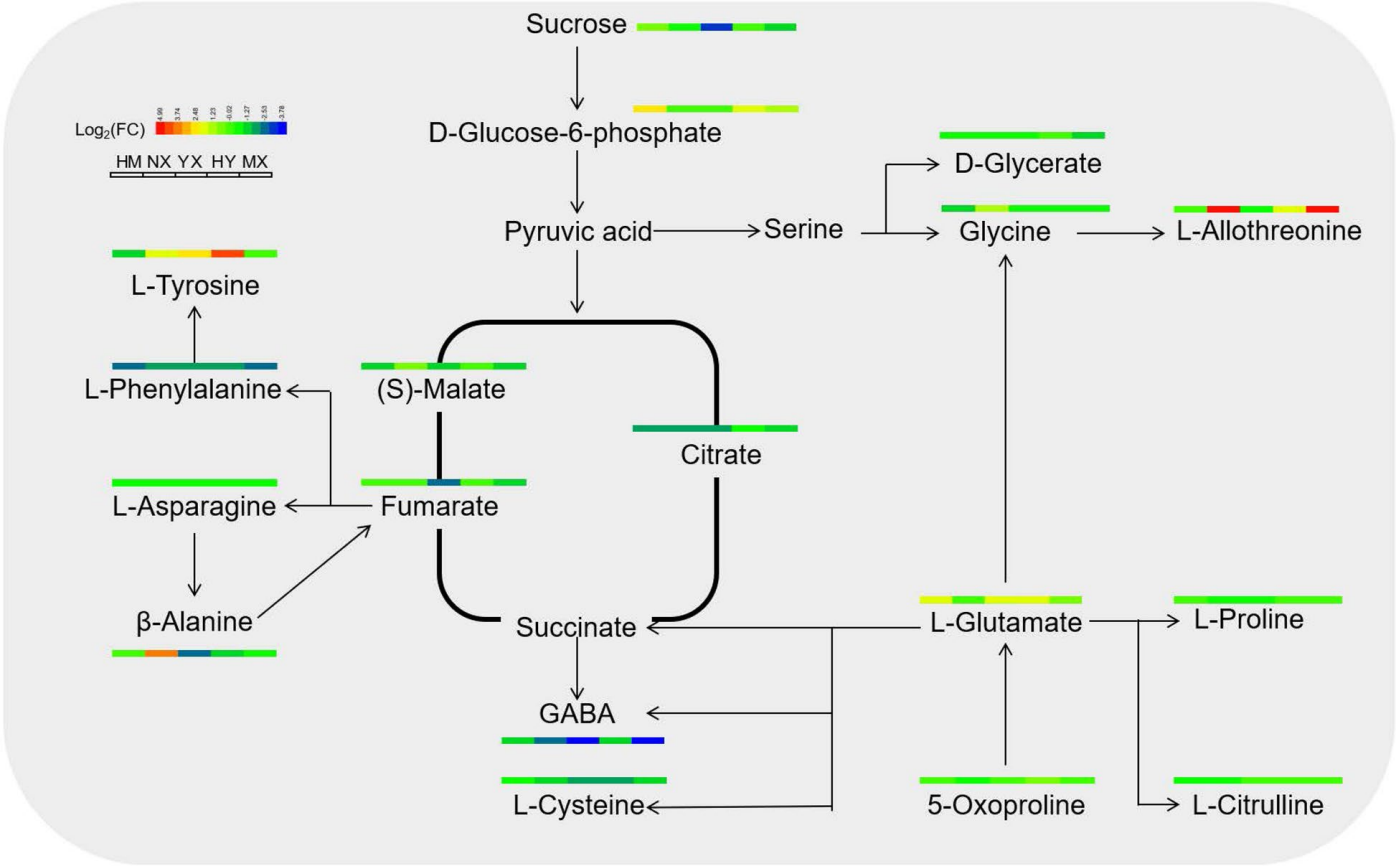
Metabolic maps of discriminating metabolites involved in potemtial targeted pathways. The potential target metabolic pathways were selected with pathway impacts of above 0.1. Log_2_(FC) stand for an estimate of the log_2_-transformed ratio of the relative content of metabolites in rice seeds of other types and CH

Our study revealed that amino acids, sugars and fatty acids played important roles in distinguishing rice seeds of CH and other varieties. In the study, metabolite profiles in CH could by discriminated from others based on PCA and PLS-DA (Figs. 3 and 4), amino acids, sugars, and fatty acids were contained in the predominated DMs (Figs. 6 and 7), and the main potential target metabolic pathways were amino acid metabolisms, and sugar metabolisms (Fig. 7; Table 1). Similarly, Sun [18] found that there were significant differences in metabolite profiles between rice seeds of different varieties (landrace and cultivated rice seeds). While differing from our research results, Feng [19] found that GC–MS-based metabolite profiles in rice seeds of different varieties did not show significant differences in the same area (Chahayang Area, Heilongjiang Province). The differences might be caused by that varieties of the seed samples in these different studies were different.

Protein, starch and lipids were the main nutritious substances of rice seeds, the synthesis of which were closely related to amino acids, sugars, and fatty acids, respectively [20]. The variety-based different in contents of amino acids, sugars, and fatty acids suggested that contents of proteins, starch and lipids were different between CH and others (Fig. 6). Rice of different varieties presented different photosynthetic characteristics and leaf nitrogen contents [21]. As the small molecule substances (e.g., amino acids, sugars, and fatty acids) required for the synthesis of proteins, starch and lipids in seeds mostly came from leaves, these might result in the significant differences of contents of amino acids, sugars, and fatty acids in rice seeds, leading to the differences in the accumulation of protein, starch and lipids (Figs. 6 and 7) [21–23].

### Amino acids played vital roles in distinguishing CH and others

The present study revealed that amino acids acted vital roles in discriminating rice seeds of different varieties, especially between CH and NX, and between CH and YX. Amino acids and amino acid derivatives were contained in the predominated DMs, especially phenylalanine and glycine (Fig. 6), and most potential target metabolic pathways, including phenylalanine metabolism, glycine, serine and threonine metabolism, and alanine, aspartate and glutamate metabolism, were amino acid metabolisms of NX and YX (Fig. 7; Table S2). Phenylalanine was essential amino acid of human, which must be obtained through dietary protein and participated in carbohydrate metabolism and fat metabolisms [24]; glycine, the simplest amino acid, was the primitive nutritional form in organisms, and participated in the synthesis of purines, porphyrins, creatine, and glyoxylate, acting as an important inhibitory neurotransmitter in the central nervous system [25, 26]. In the study, the content of phenylalanine in NX was more than twice that in CH, and glycine content of CH was more than 93 times that of YX. These suggested that, compared to CH, rice seeds of NX were more conducive to metabolism of carbohydrate and fat, and healthy growth maintenance of the human body, and compared to YX, rice seeds of CH was more suitable as potential glycine supplement.

Besides, the main storage protein glutenin and gliadin in CH might be lower than those both in YX and NX. Glutamic acid and aspartic acid hold high proportions in the synthesis of glutenin and gliadin, respectively, and were the foundation of the two main storage protein [27–29]. In the present study, glutamate and aspartate upregulated in rice seeds both of NX and YX (Fig. 6; Table S1). These suggested that content of glutenin and gliadin in rice seeds of NX and YX were higher than those of CH. Similarly, Shi [30] sampled rice seeds of different varieties, and found that rice seeds of different varieties presented different content of storage proteins.

### Sugars played vital roles in distinguishing different varieties

The study identified that sugars acted vital roles in discriminating rice seeds of different varieties, especially between CH and HM, between CH and HY, between CH and NX, and between CH and MX. The proportion of sugars and sugar derivatives were high in the predominated DMs, especially lactulose, and 1,5-anhydroglucitol (Fig. 6; Table S1). Lactulose was a functional oligosaccharide, and an effective proliferation factor for bifidobacteria. It had special physiological functions such as bacterial proliferation, lowering cholesterol in the blood, improving blood lipids, and promoting calcium absorption [31, 32]. In the study, lactulose contents of HM, HY, and MX were up-regulated. This suggested that rice seeds of HM, HY, and MX were more suitable as potential lactulose supplement, compared to those of CH.

Besides, rice seeds of CH might be more suitable for the patients with diabetes, compared to those of NX. 1,5-anhydroglucitol content in seeds of NX was more than 13 times that of CH (Fig. 6; Table S1). 1,5-anhydroglucitol, one of the main polyol sugars in the human body, was mainly derived from food, and could be affected by dietary habit [33]. 99.9% of 1,5-anhydroglucitol in the body was reabsorbed by the kidneys [34]. When the blood sugar content was higher than the renal threshold in patients with diabetes, it would competitively inhibit the reabsorption of 1,5-AG in kidney, resulting in the increased excretion of 1,5-AG in urine and decreased content in serum [35]. The content of 1,5-anhydroglucitol in serum could be used as indicators for short-term blood glucose monitoring [36]. Eating food with a higher percentage of 1,5-anhydroglucitol, patients with diabetes might enhance the kidney reabsorption of 1,5-anhydroglucitol, and interfere with blood sugar monitoring [35].

## Conclusions

Rice seeds of different varieties hold different metabolic profiles. In the study, we would reveal the differences among the six varieties via comparing the predominant metabolites (e.g., amino acids, sugars, and organic acids), especially the predominant DMs.

1. Compared to CH, 18, 42, 45, 30 and 28 DMs were screened in HM, NX, YX, HY, and MX, respectively, and the number of both up DMs and down DMs were different between different groups. Most of the DMs were up regulated in NX, while most were down regulated in YX. This revealed that metabolic profiles were different in rice seeds of different varieties.
2. Among all the metabolites, organic acids, amino acids, and sugars showed higher proportions in rice seeds of all different varieties, and contributed more to the separation between CH and the others. And amino acid metabolisms accounted for the most among the potential target metabolic pathways, which involved starch and sucrose metabolism. This implied that contents of proteins, starch, and lipids might be different in seeds of CH and the others.
3. Compared to seeds of CH, amino acids with the greatest changes in content were phenylalanine of NX (above twice), and glycine of YX (above 93 times), and glutamate and aspartate up-regulated in seeds both of NX and YX. Due to the biological functions of these amino acids, these indicated that compared to CH, rice seeds of NX were more conducive to metabolism of carbohydrate and fat, and healthy growth maintenance in the human body, and rice seeds of YX was not suitable as potential glycine supplement, and content of glutenin and gliadin in seeds of NX and YX were higher than those of CH.
4. Compared to seeds of CH, lactulose contents in seeds of HM, HY, and MX were up-regulated, and 1,5-anhydroglucitol (above 13 times) showed the greatest changes in content of NX. Because of the biological functions of the two sugars, rice seeds of HM, HY, and MX were more suitable as potential lactulose supplement, compared to those of CH, and rice seeds of NX might be not suitable for the patients with diabetes.

### Supplementary Information

Supplementary Material 1.

Supplementary Material 2.

Supplementary Material 3.

Supplementary Material 4.

## Data Availability

The datasets used and/or analysed during the current study are available from the corresponding author on reasonable request.

## Abbreviations

GC–MS: Gas Chromatography-Mass Spectrometry
GC-TOF–MS: Gas Chromatography Time-Of-Flight Mass Spectrometry
CH: Changhui 871
HM: Huaxiang madi
NX: Nongxiang 39
YX: Yahe xiang
HY: Huaxiang Yousi
MX: Meixiangzhan 2
ANOVA: Analysis of Variance
PCA: Principal Component Analysis
PLS-DA: Partial least squares discrimination analysis
VIP: The variable importance in projection
DMs: Discriminating metabolites
KEGG: Kyoto Encyclopedia of Genes and Genomes
TCA cycle: Citrate cycle
