# Supplementary figures and images for: Non-targeted GC–MS metabolomics-based differences in Indica rice seeds of different varieties
Source: BMC Plant Biol. 2024 Jun 8;24:519. doi: 10.1186/s12870-024-05255-6 (PMC11162567; doi:10.1186/s12870-024-05255-6)

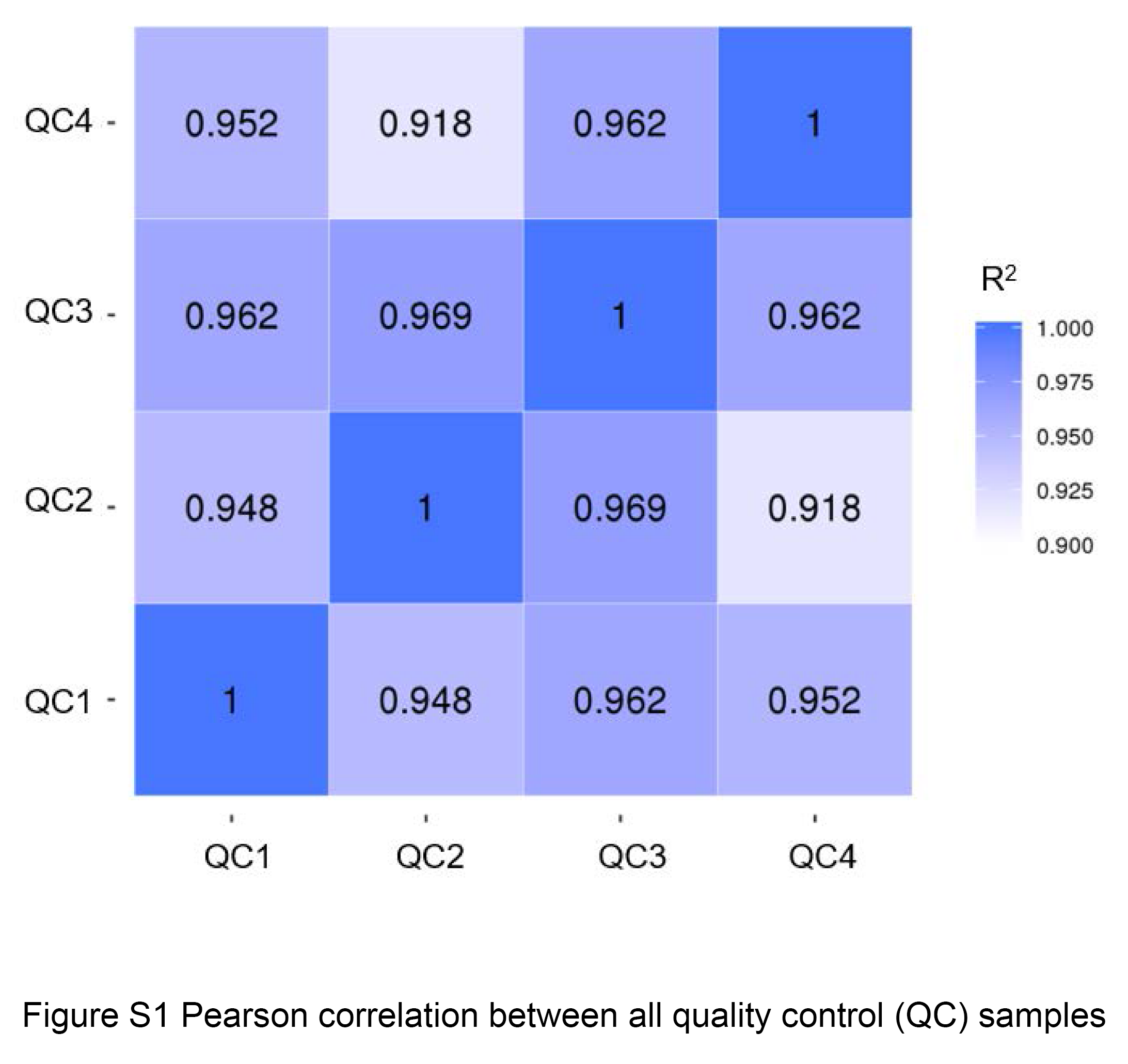

Supplement: Supplementary file 1 — Supplementary Material 1. [file 12870_2024_5255_MOESM1_ESM.tiff]

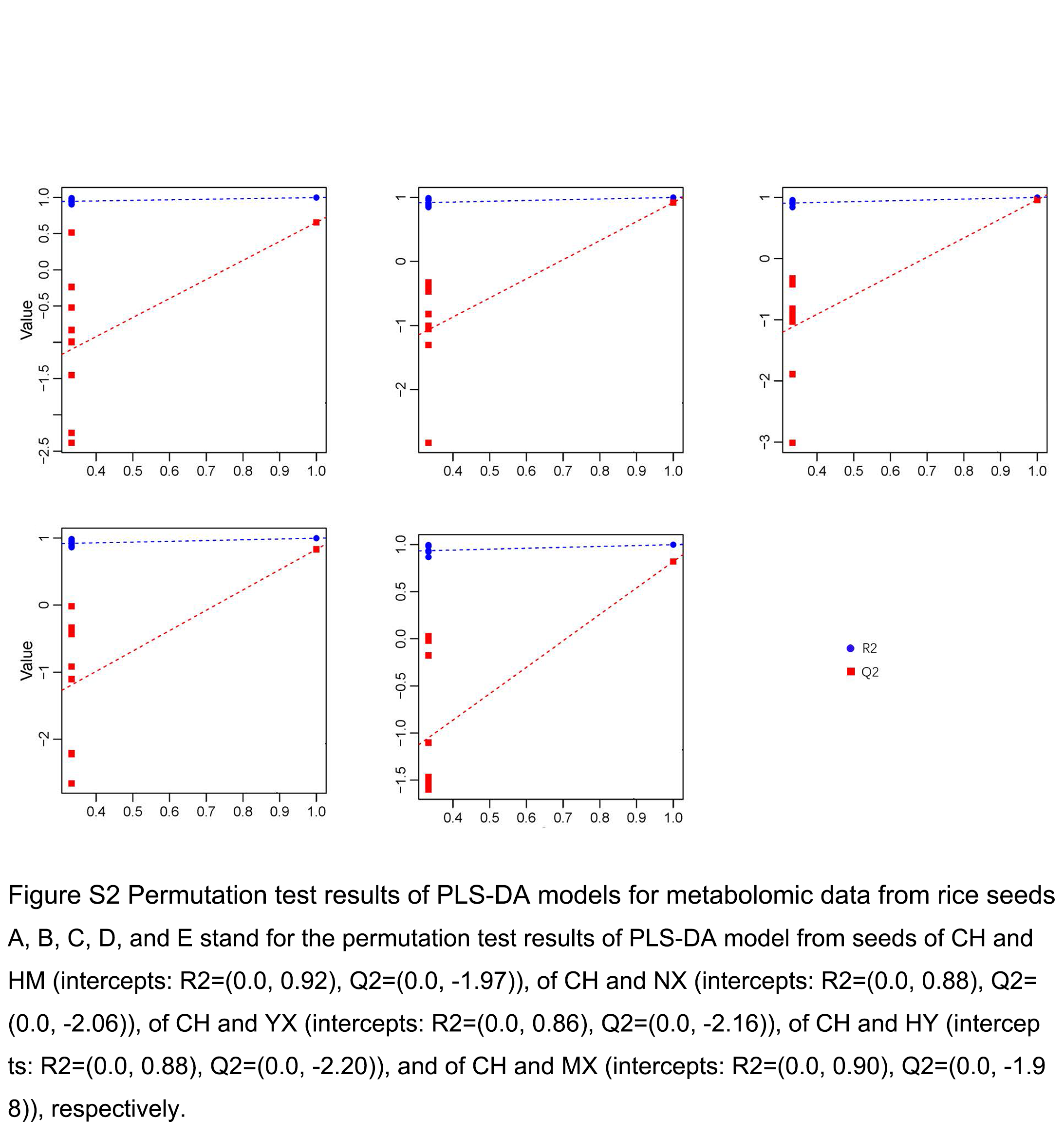

Supplement: Supplementary file 2 — Supplementary Material 2. [file 12870_2024_5255_MOESM2_ESM.tiff]
