# Supplementary material for: Non-targeted GC–MS metabolomics-based differences in Indica rice seeds of different varieties
Source: BMC Plant Biol. 2024 Jun 8;24:519. doi: 10.1186/s12870-024-05255-6 (PMC11162567; doi:10.1186/s12870-024-05255-6)
Supplement: Supplementary file 3 — Supplementary Material 3. [file 12870_2024_5255_MOESM3_ESM.docx]

| ID | Metabolites | RT (min) | CH | HM | NX | YX | HY | MX |
| --- | --- | --- | --- | --- | --- | --- | --- | --- |
| Com_81 | 2-amino-2-methylpropane-1,3-diol 1 | 6.92 | 2253464.25±177348.33 | 2624832.16±196922.91 | 2377985.17±156239.70 | 2328957.63±167347.04 | 2372217.47±125587.76 | 2302896.09±153125.23 |
| Com_6 | Lactamide 1 | 7.06 | 23793.69±1692.20 | 12559.67±3431.86 | 32867.69±3414.88 | 19711.94±628.88 | 31321.81±1889.43 | 20187.24±1673.00 |
| Com_70 | Linoleic acid methyl ester | 7.18 | 457328.89±4763.77 | 399815.49±60975.56 | 574521.10±43688.40 | 234990.92±26632.60 | 364498.07±22458.10 | 359660.45±27825.71 |
| Com_90 | Carbobenzyloxy-L-leucine degr1 | 7.20 | 533024.79±17852.23 | 716389.97±139721.36 | 614880.99±33421.70 | 561759.22±15774.20 | 516260.56±5949.50 | 532519.97±9045.94 |
| Com_109 | creatine degr | 7.41 | 390458.96±31842.31 | 442755.08±58650.87 | 314801.17±16861.84 | 172850.68±7163.84 | 313536.42±25103.83 | 197491.90±6582.44 |
| Com_60 | Methyl Phosphate | 7.79 | 7822227.69±200282.15 | 9604623.91±697539.09 | 8660058.68±505095.73 | 1272378.99±83234.18 | 13394515.40±492180.05 | 4074587.69±150673.59 |
| Com_79 | 2-Monoolein | 7.91 | 504801.82±23947.75 | 542183.50±45419.11 | 536292.74±22675.79 | 478386.96±25053.66 | 539306.03±18320.42 | 502752.87±19170.19 |
| Com_86 | 4-hydroxyphenylacetic acid | 8.06 | 706980.88±56257.13 | 1037013.64±221429.59 | 2006792.94±272866.10 | 100706.21±13261.63 | 1026724.63±134293.58 | 336368.50±53199.95 |
| Com_62 | Digitoxose 2 | 8.10 | 13412.06±714.17 | 7356.18±2314.57 | 8187.43±3161.54 | 14599.78±1242.41 | 5084.77±0.07 | 13539.31±2383.21 |
| Com_46 | lysine | 8.17 | 77078.86±19962.47 | 76359.88±3773.43 | 80557.06±22283.76 | 75691.73±6662.44 | 84048.40±14452.82 | 77640.63±19350.35 |
| Com_106 | Tagatose 1 | 8.33 | 8665420.43±1253234.13 | 10471635.44±561745.59 | 8544552.41±1290032.86 | 9097278.72±1607510.92 | 10548099.93±133976.95 | 9128196.27±1350645.33 |
| Com_152 | melibiose 1 | 8.35 | 95924.32±42329.34 | 76594.50±30712.30 | 76400.02±50702.82 | 95460.71±42422.35 | 61968.55±44860.97 | 98902.26±41310.15 |
| Com_135 | Ribonic acid, γ-lactone | 8.43 | 1034214.16±57737.45 | 1114173.00±189329.29 | 527772.49±42374.83 | 148540.99±19361.26 | 751697.15±95679.56 | 550601.44±27878.85 |
| Com_69 | lactose 1 | 8.56 | 66061.90±10318.19 | 73823.66±6447.13 | 80369.55±14328.83 | 75940.53±10055.96 | 66771.30±4075.70 | 61659.95±7658.50 |
| Com_25 | L-cysteine | 8.59 | 10761.74±7102.53 | 9081.89±2704.56 | 5053.83±1287.07 | 3790.95±0.07 | 3790.53±0.23 | 5591.20±1834.50 |
| Com_80 | 2-ketoadipate 3 | 8.59 | 214959.58±40194.22 | 227201.07±11017.90 | 264626.44±48868.32 | 217233.08±27727.25 | 211372.73±17156.61 | 195300.55±27626.13 |
| Com_88 | asparagine 1 | 8.66 | 15295.15±11363.13 | 21867.75±9307.61 | 31652.75±28029.27 | 5523.02±1406.49 | 14812.88±5517.70 | 11343.73±7337.06 |
| Com_68 | maltose | 8.67 | 256752.51±44541.43 | 473281.98±19619.85 | 507354.03±55737.68 | 269011.83±20108.49 | 322064.89±14882.49 | 296949.08±59414.11 |
| Com_39 | 2-hydroxybutanoic acid | 8.70 | 8399.90±988.89 | 9521.81±1221.96 | 11312.23±1047.34 | 8496.68±1037.21 | 7938.31±777.85 | 9985.27±1832.55 |
| Com_27 | fumaric acid | 8.76 | 26821.73±3768.56 | 31663.81±2944.72 | 30779.71±4317.54 | 4922.65±669.59 | 32693.10±699.06 | 16928.04±2366.73 |
| Com_16 | oxamic acid | 8.84 | 709425.85±87525.89 | 760172.16±90525.61 | 1503601.01±163037.72 | 1026266.09±45477.90 | 852813.91±17431.93 | 1399238.57±141227.64 |
| Com_38 | sucrose | 9.05 | 20849.21±498.89 | 40126.99±6287.04 | 20050.30±705.83 | 2924.45±744.74 | 27034.46±495.25 | 10428.05±1111.27 |
| Com_67 | glycocyamine 3 | 9.22 | 69209.24±7863.12 | 75946.34±11209.52 | 66668.10±10252.38 | 76293.90±12757.88 | 65824.02±4045.29 | 76118.03±12266.49 |
| Com_94 | Glucosaminic acid | 9.25 | 139801.86±7933.90 | 139139.75±5240.18 | 142178.87±11363.41 | 156395.87±8695.37 | 126742.69±1990.29 | 148302.50±14174.80 |
| Com_51 | Itaconic acid | 9.29 | 69214.02±2526.24 | 78688.24±3305.38 | 92976.45±7857.71 | 66590.15±3413.49 | 75962.32±1664.80 | 70130.38±2588.09 |
| Com_4 | 1,5-Anhydroglucitol | 9.37 | 1090037.64±38261.46 | 1181614.15±62978.47 | 7594301.02±1328911.44 | 375187.15±18706.06 | 829907.50±63594.03 | 1198579.22±85434.74 |
| Com_17 | N-Acetyl-β-D-mannosamine 4 | 9.57 | 38154.86±2243.75 | 31255.83±2908.71 | 77081.37±5196.50 | 33875.60±6569.94 | 41812.02±11535.05 | 41006.14±3634.87 |
| Com_63 | 1-Hexadecanol | 9.67 | 66194.78±3837.69 | 67719.72±2059.15 | 76950.61±2597.81 | 81837.88±3809.18 | 64983.08±3614.55 | 72978.93±6388.19 |
| Com_74 | ribose | 9.80 | 64250.53±2901.60 | 61889.93±3780.96 | 78358.34±1910.54 | 83876.03±3701.47 | 64983.22±1021.34 | 78270.42±1532.45 |
| Com_65 | Neohesperidin | 9.81 | 56327.62±2422.15 | 51272.67±1109.15 | 72173.50±2326.20 | 27216.17±15519.47 | 59376.18±674.58 | 55453.84±753.70 |
| Com_133 | caffeic acid | 9.86 | 173863.64±9140.86 | 81477.54±53185.14 | 65886.64±37300.14 | 140509.54±8078.21 | 206984.77±13053.94 | 149737.23±6011.79 |
| Com_85 | androsterone 1 | 9.89 | 23520.82±1217.29 | 39567.81±4014.38 | 10905.10±2989.27 | 11668.11±533.54 | 27550.24±1742.37 | 15362.16±517.55 |
| Com_72 | cycloleucine 2 | 9.91 | 82427.51±2940.42 | 83885.51±3856.50 | 102315.70±915.04 | 85992.73±3829.92 | 96193.68±3020.09 | 86733.23±1280.43 |
| Com_50 | gluconic acid 1 | 9.97 | 43543.40±2039.97 | 36304.77±4947.31 | 67484.57±2480.14 | 23250.31±897.43 | 55347.06±1982.13 | 39436.99±8365.88 |
| Com_5 | Allantoic acid 3 | 10.08 | 907638.91±171100.67 | 1079416.83±361039.99 | 1625606.20±205783.67 | 1026546.69±184065.03 | 1137115.00±145616.30 | 1291890.96±255641.33 |
| Com_75 | N-Acetyl-L-leucine 1 | 10.15 | 7348732.61±311891.45 | 7758675.64±914973.61 | 6016403.62±554165.59 | 3761616.81±193489.25 | 6147854.03±298176.84 | 5822955.41±181684.04 |
| Com_146 | serine 1 | 10.18 | 9467452.93±311520.83 | 7528825.64±345027.23 | 5962689.87±232007.23 | 2400510.23±264194.18 | 7122177.19±69944.29 | 3772238.21±160797.29 |
| Com_202 | 2-hydroxypyridine | 10.18 | 14654.36±9097.79 | 72224.35±41307.19 | 190810.29±58599.10 | 5725.15±0.13 | 121698.43±29076.26 | 76033.29±71633.92 |
| Com_95 | 2-Deoxyerythritol | 10.28 | 343520.24±18609.66 | 371371.86±27177.22 | 348848.77±21719.01 | 362831.89±15825.85 | 329503.99±10158.28 | 340010.08±31711.88 |
| Com_15 | Phenylphosphoric acid | 10.37 | 74154.54±4230.83 | 71360.72±10960.58 | 98277.20±9731.98 | 57717.59±3579.14 | 76639.97±6159.45 | 73955.52±2861.58 |
| Com_206 | naringenin 1 | 10.39 | 39580.07±1563.58 | 31128.86±5348.04 | 58203.22±5538.63 | 11062.32±0.26 | 24466.52±1246.25 | 37111.47±2335.31 |
| Com_29 | Cysteinylglycine 1 | 10.45 | 566292.68±8187.38 | 585319.31±149726.69 | 3920705.80±799192.49 | 198833.83±16999.08 | 351572.75±30474.86 | 688294.16±55828.58 |
| Com_203 | adrenaline 1 | 10.53 | 2001342.94±54162.18 | 1422662.36±462233.12 | 8888578.52±2183046.90 | 439711.67±39442.03 | 920049.23±52324.91 | 973119.45±112465.16 |
| Com_41 | oxalic acid | 10.57 | 39951.72±5261.82 | 43822.88±13654.82 | 78403.35±11656.21 | 21065.44±4746.42 | 11957.76±0.14 | 11958.27±0.07 |
| Com_8 | 4-Methyl-5-thiazolethanol | 10.63 | 396703.98±51491.88 | 533345.57±259266.21 | 1561634.10±91623.29 | 76794.74±21565.13 | 576139.95±89334.49 | 231707.87±41681.25 |
| Com_97 | Uridine monophosphate | 10.69 | 22206.40±2574.97 | 17466.57±5783.49 | 18493.54±1352.23 | 24163.62±3089.32 | 18325.10±796.65 | 19599.10±1544.40 |
| Com_71 | 4-hydroxybutyrate | 10.74 | 12802.40±1919.37 | 9347.00±3082.50 | 10880.03±2218.78 | 12745.34±425.22 | 6279.62±2876.31 | 6752.45±3358.10 |
| Com_213 | α-D-glucosamine 1-phosphate | 10.75 | 402970.53±64580.27 | 872439.58±241418.96 | 413116.51±63640.10 | 238085.44±10032.75 | 556244.89±40746.67 | 608793.41±25383.83 |
| Com_220 | 3-Methylamino-1,2-propanediol 1 | 10.76 | 50995.60±8240.43 | 86752.59±24129.45 | 69544.04±9588.96 | 23844.40±6281.43 | 49501.06±1526.85 | 62122.90±1486.86 |
| Com_32 | N(epsilon)-Trimethyllysine | 10.90 | 330638.21±157669.98 | 368983.18±56881.45 | 389176.22±72494.62 | 533295.69±10837.32 | 465787.74±28476.88 | 529842.80±45405.03 |
| Com_58 | citrulline 1 | 10.92 | 157665.80±1497.64 | 127614.31±26824.75 | 131488.25±27645.17 | 162349.51±11925.18 | 173812.65±14323.41 | 169148.73±17322.56 |
| Com_127 | N-cyclohexylformamide 1 | 10.93 | 119603.04±18074.54 | 182834.16±39747.79 | 36913.08±10577.78 | 23537.40±6622.14 | 149207.56±19029.67 | 91000.56±6323.61 |
| Com_87 | trans-sinapinic acid | 11.06 | 91187.86±11644.08 | 163919.89±38203.03 | 47995.96±12243.07 | 56184.71±3978.33 | 124899.78±9756.90 | 227426.89±14630.51 |
| Com_35 | arbutin | 11.11 | 12446.12±4724.84 | 5384.73±0.30 | 50800.28±11255.15 | 30391.90±2851.62 | 59356.46±10004.94 | 5384.91±0.11 |
| Com_2 | Abietic Acid 1 | 11.24 | 27762.37±955.85 | 130251.20±31005.23 | 10223.85±4360.90 | 23335.79±1658.27 | 52316.22±9092.97 | 135284.02±4440.94 |
| Com_20 | glucose 1 | 11.34 | 21947.79±8031.25 | 6188.50±0.14 | 6189.00±0.07 | 21744.70±8275.39 | 6188.54±0.25 | 26768.36±10614.62 |
| Com_218 | palmitic acid | 11.34 | 1308120.46±71765.72 | 861003.69±192305.79 | 5838143.33±1659532.37 | 553051.15±48535.19 | 582758.63±75095.97 | 1691664.16±128478.70 |
| Com_89 | asparagine 4 | 11.40 | 47425.27±5608.75 | 31773.59±5023.48 | 39109.72±8214.37 | 33378.33±573.87 | 40689.94±9083.73 | 43871.08±1222.69 |
| Com_143 | phytosphingosine 1 | 11.42 | 164699.43±14697.65 | 92134.37±23756.09 | 34352.45±12548.85 | 75703.55±6265.93 | 44087.16±4361.36 | 120980.81±10945.18 |
| Com_125 | Glucoheptonic acid 3 | 11.51 | 288824.77±46691.75 | 135911.18±36303.44 | 737542.99±132026.57 | 272364.48±24292.25 | 636734.17±35660.27 | 217774.78±36253.65 |
| Com_82 | L-homoserine 1 | 11.54 | 59771.11±8853.38 | 44231.98±12691.62 | 44363.66±34989.20 | 57205.40±873.84 | 47639.43±7597.76 | 54723.25±6982.56 |
| Com_102 | Sophorose 2 | 11.58 | 12818.23±0.26 | 12818.00±0.14 | 12818.51±0.07 | 12818.08±0.23 | 12818.09±0.26 | 12818.03±0.12 |
| Com_33 | epsilon-Caprolactam | 11.67 | 281382.15±31045.58 | 219013.47±62094.17 | 1403023.11±570022.29 | 107240.31±16894.77 | 187669.95±11567.12 | 275225.76±16735.66 |
| Com_116 | glycine 1 | 11.69 | 73611.38±7442.60 | 37130.68±22895.47 | 184424.10±83124.41 | 50238.31±4067.25 | 64742.28±5100.78 | 47232.01±19477.58 |
| Com_114 | oleic acid | 11.77 | 11369.35±643.88 | 8791.99±3193.76 | 7017.45±3013.37 | 8390.54±2864.32 | 10134.20±937.75 | 8615.87±957.02 |
| Com_21 | lactulose 1 | 11.86 | 3750.44±1439.65 | 3542.29±1143.27 | 2896.53±1450.56 | 2857.50±789.81 | 4956.93±713.90 | 4693.32±137.72 |
| Com_76 | salicin | 11.93 | 10553.45±2636.41 | 7655.28±2835.17 | 6132.83±669.10 | 9547.77±968.31 | 8763.28±3485.68 | 9997.21±3908.74 |
| Com_34 | uridine 2 | 11.96 | 34620.95±2539.99 | 20168.26±6058.11 | 45551.78±14277.16 | 59879.83±4067.92 | 13581.94±4660.63 | 32657.23±5392.27 |
| Com_59 | glycolic acid | 12.03 | 214368.98±43220.75 | 150695.26±53155.52 | 134595.98±24237.61 | 176727.57±32053.24 | 211275.45±29894.83 | 203343.84±46642.94 |
| Com_7 | allose 1 | 12.21 | 73838.28±393.98 | 38277.53±10376.44 | 65214.71±18704.78 | 97466.61±5213.55 | 35757.45±3681.93 | 103946.50±3470.96 |
| Com_210 | 3-Aminoisobutyric acid 1 | 12.50 | 9785.10±0.30 | 9785.28±0.11 | 19798.36±5837.75 | 9785.36±0.20 | 27881.62±9481.61 | 18047.24±4480.12 |
| Com_49 | Dehydroabietic Acid | 12.50 | 67496.85±10896.03 | 63620.18±17717.47 | 57179.54±16484.32 | 50846.24±2485.14 | 79946.08±5134.58 | 75719.96±3617.01 |
| Com_209 | O-acetylserine 2 | 12.51 | 14613.33±1692.35 | 17097.14±4658.05 | 16389.77±3132.34 | 6530.56±1418.08 | 11743.61±4060.39 | 11302.89±1039.68 |
| Com_66 | 1,3-Cyclohexanedione 1 | 12.55 | 32534.62±3225.13 | 15756.75±5039.72 | 19184.59±11503.89 | 26399.84±1829.68 | 29936.49±5143.94 | 35115.38±4098.48 |
| Com_45 | 2-hydroxy-3-isopropylbutanedioic acid | 12.65 | 340629.93±50264.32 | 301040.54±81772.20 | 310416.23±64749.97 | 326743.75±46577.11 | 353219.08±21387.79 | 340735.34±49549.74 |
| Com_101 | N-α-Acetyl-L-ornithine 3 | 12.66 | 27227.41±0.26 | 27227.18±0.14 | 27227.69±0.07 | 27227.26±0.23 | 27227.26±0.26 | 27227.21±0.12 |
| Com_64 | 3-Hydroxypropionic acid 1 | 12.69 | 35569.48±2870.03 | 25135.01±5204.83 | 40320.77±10021.72 | 34899.66±937.35 | 43244.63±2984.11 | 55219.27±3052.05 |
| Com_30 | maltotriose 1 | 12.77 | 57993.44±14953.55 | 62760.74±27355.36 | 126581.08±25836.19 | 9547.08±1797.94 | 75458.90±20958.49 | 30960.88±7833.91 |
| Com_207 | O-methylthreonine 2 | 12.82 | 7902.50±1794.65 | 10540.73±3819.16 | 15954.24±7971.93 | 6609.24±2180.89 | 14460.46±5093.09 | 33261.40±3636.46 |
| Com_12 | D-Glyceric acid | 12.93 | 20064.27±8623.46 | 19233.83±3506.68 | 14518.30±4352.13 | 14309.07±129.01 | 23389.69±1231.32 | 12406.51±4698.35 |
| Com_107 | D-(glycerol 1-phosphate) | 13.00 | 457921.64±10644.20 | 1773465.45±252805.58 | 296675.13±83140.02 | 322868.48±16172.00 | 1848216.40±77959.97 | 1504524.83±49974.69 |
| Com_55 | Ergosterol | 13.13 | 39604.10±1164.79 | 94097.63±4716.66 | 20557.11±4980.23 | 7576.38±732.52 | 40340.99±3378.83 | 50601.65±3134.95 |
| Com_61 | 3-hydroxy-L-proline 2 | 13.22 | 1420347.71±22601.52 | 745110.73±43117.96 | 5710114.55±1579235.81 | 1015925.26±52685.64 | 3530893.00±46169.02 | 825880.34±11368.29 |
| Com_216 | Gentiobiose 1 | 13.29 | 28298.10±0.30 | 103598.78±1788.82 | 59784.63±18820.67 | 28298.47±0.13 | 28298.36±0.20 | 28298.12±0.27 |
| Com_149 | maleic acid | 13.30 | 64584.51±3308.92 | 118104.04±5626.05 | 27561.99±5413.31 | 37757.65±896.09 | 58022.69±1124.55 | 44351.64±3127.17 |
| Com_19 | stearic acid | 13.41 | 2215059.39±51219.28 | 1770058.44±267706.29 | 3575672.63±787262.49 | 2107027.56±90462.57 | 1017472.14±72499.25 | 2363948.94±31875.59 |
| Com_204 | phosphate | 13.45 | 4327058.51±88209.27 | 4393410.45±576905.09 | 30484705.03±4101806.21 | 9988532.59±751741.01 | 5293705.81±362716.36 | 10681546.02±847086.96 |
| Com_26 | D-Arabitol | 13.54 | 3534187.52±886555.00 | 3241311.47±967059.72 | 1232780.85±110803.93 | 103016.03±27951.10 | 2472058.00±304118.11 | 1714715.05±280657.85 |
| Com_52 | uracil | 13.64 | 243856.46±8481.81 | 293086.05±30809.50 | 364668.06±121549.55 | 266530.12±12609.24 | 230028.80±4824.11 | 233217.89±15042.40 |
| Com_13 | Galactonic acid | 13.84 | 47015.11±2329.46 | 39978.81±3109.42 | 191909.70±30807.94 | 64566.43±1017.41 | 34974.57±1177.87 | 57764.47±373.84 |
| Com_208 | Dehydroepiandrosterone | 13.86 | 96075.92±860.76 | 113858.41±12614.25 | 332359.03±57036.66 | 185824.11±2129.04 | 65714.29±3695.57 | 133927.24±1690.75 |
| Com_112 | proline | 14.02 | 44363.27±2730.77 | 44104.67±3798.13 | 36866.39±7341.14 | 43024.47±4540.94 | 47848.70±876.83 | 47464.20±2728.74 |
| Com_98 | glutamic acid | 14.13 | 3833.99±0.26 | 14776.11±1660.80 | 3834.26±0.13 | 17044.80±223.67 | 14440.47±741.30 | 8429.32±428.75 |
| Com_124 | Glutaric Acid | 14.38 | 394104.52±17067.27 | 273403.67±16756.75 | 486343.61±80817.56 | 228154.81±11299.46 | 218612.46±61874.97 | 243963.43±25230.81 |
| Com_92 | 4-HYDROXYPYRIDINE | 14.44 | 25434.30±395.28 | 23528.11±3382.99 | 100108.41±13384.62 | 23323.11±2201.23 | 14385.40±2281.48 | 31805.24±1885.52 |
| Com_99 | azelaic acid | 14.59 | 10533.09±0.30 | 10533.27±0.11 | 10533.38±0.22 | 10533.08±0.25 | 10533.14±0.25 | 10533.21±0.09 |
| Com_200 | D-Talose 2 | 14.60 | 130264.52±7966.93 | 185860.32±13910.73 | 1363454.62±189650.95 | 392490.19±16160.74 | 216083.03±14964.77 | 428714.65±16780.79 |
| Com_9 | β-Alanine 1 | 14.70 | 230360.21±12817.61 | 261798.02±27168.86 | 3792978.04±595636.57 | 53722.65±4852.56 | 144955.52±9209.58 | 219845.12±9944.04 |
| Com_37 | xylitol | 14.83 | 141096.97±13652.52 | 85575.40±18832.90 | 119575.04±36394.00 | 47923.49±5309.64 | 129110.96±1997.49 | 89492.72±2363.03 |
| Com_197 | Phenyl β-D-glucopyranoside | 14.92 | 156336.10±5965.70 | 120568.79±10162.75 | 516034.33±42206.44 | 12296.67±3583.83 | 31265.59±1810.30 | 374423.29±26951.28 |
| Com_1 | oxoproline | 14.92 | 7609.00±4291.42 | 8280.23±4975.36 | 6389.62±3048.70 | 11062.32±4084.51 | 13988.72±8341.06 | 11186.73±4916.99 |
| Com_212 | lactic acid | 14.97 | 52889.27±599.33 | 58757.91±4814.70 | 77002.19±1892.59 | 29581.32±1697.83 | 32029.54±2155.73 | 91233.56±3709.34 |
| Com_113 | Levoglucosan | 15.02 | 6810.14±1038.71 | 3157.19±804.02 | 9119.84±343.54 | 2368.17±0.26 | 3276.33±925.12 | 2368.36±0.22 |
| Com_22 | Threitol | 15.05 | 258750.01±5291.91 | 217537.52±11041.25 | 335144.08±3882.10 | 110740.78±3830.45 | 169010.65±10382.57 | 312193.86±6071.86 |
| Com_130 | 1-Monopalmitin | 15.18 | 570732.36±60854.97 | 379054.52±20780.29 | 6045811.98±456417.01 | 625146.19±21431.38 | 2322596.25±112219.72 | 394736.03±29277.36 |
| Com_138 | shikimic acid | 15.30 | 53416.81±1041.38 | 60469.86±28041.57 | 66764.79±11736.16 | 14849.00±749.53 | 45256.89±3333.14 | 20199.21±2487.55 |
| Com_211 | Maleamate 2 | 15.34 | 14906.55±0.26 | 33414.90±1530.71 | 47440.22±4317.22 | 32835.99±1666.90 | 57251.19±3421.90 | 37308.76±359.26 |
| Com_47 | 2-Monopalmitin | 15.48 | 22808.24±645.99 | 13866.50±1905.10 | 75849.34±13655.76 | 34250.55±2271.35 | 14988.23±2142.62 | 24271.85±1081.35 |
| Com_165 | palmitoleic acid | 15.54 | 685210.59±8959.40 | 1155468.38±36258.81 | 312858.05±68162.30 | 56058.98±2150.17 | 1049509.21±61868.00 | 52965.89±5025.73 |
| Com_194 | Prostaglandin E2 2 | 15.56 | 10399.86±0.30 | 10400.04±0.11 | 60964.88±9857.11 | 10400.15±0.22 | 10399.86±0.25 | 10399.91±0.25 |
| Com_183 | ferulic acid | 15.70 | 57639.47±53724.76 | 30381.99±23497.79 | 344802.02±182603.32 | 65430.28±61662.34 | 42158.32±27326.62 | 118892.72±8865.79 |
| Com_136 | Aminomalonic acid | 15.76 | 50692.67±21586.07 | 67914.16±2891.71 | 43729.31±21906.90 | 63483.83±910.41 | 65145.03±586.99 | 62546.30±1031.04 |
| Com_126 | Capric Acid | 15.78 | 33816.75±26233.38 | 16678.20±8771.56 | 31017.50±23381.26 | 19390.85±11535.00 | 17030.80±9130.80 | 17144.43±9246.60 |
| Com_221 | β-Mannosylglycerate 1 | 15.91 | 75239.76±5691.26 | 54257.78±5902.10 | 275103.32±75322.68 | 110437.47±7948.99 | 52689.45±5118.91 | 64696.86±9366.91 |
| Com_195 | aspartic acid 1 | 15.94 | 19264.72±4153.18 | 11184.02±0.30 | 322068.42±102921.19 | 22066.38±6107.92 | 68166.50±8179.98 | 20854.89±4992.28 |
| Com_180 | Purine riboside | 16.08 | 324649.20±7046.81 | 137978.53±17032.58 | 210719.09±61044.24 | 12530.27±2307.88 | 15794.48±874.62 | 10691.77±1164.41 |
| Com_36 | Glucose-1-phosphate | 16.16 | 2232017.44±42296.59 | 1373916.37±68204.00 | 5268185.73±605004.26 | 1485421.06±31989.65 | 659129.86±579252.58 | 1861134.65±21067.00 |
| Com_77 | cis-gondoic acid | 16.19 | 3777043.70±19587.50 | 1880206.90±47927.24 | 5216096.14±291639.13 | 2260800.50±59753.69 | 3329064.29±86997.78 | 1864111.35±9912.06 |
| Com_214 | Myristic Acid | 16.28 | 11275.85±9601.60 | 11879.02±11518.92 | 45340.30±23361.31 | 26655.71±1959.72 | 59400.84±2498.05 | 26744.15±13531.83 |
| Com_215 | 2-methylfumarate | 16.36 | 45950.73±3553.85 | 84305.75±4325.91 | 4503.42±0.26 | 7889.51±1758.28 | 45922.17±2645.77 | 23455.63±725.13 |
| Com_150 | DL-Anabasine 1 | 16.54 | 4426.20±2011.74 | 5597.54±1767.76 | 8563.35±3245.71 | 3268.83±832.20 | 5899.29±497.78 | 6191.37±2274.52 |
| Com_3 | D-erythro-sphingosine 2 | 16.59 | 3369.36±0.30 | 6819.51±3515.12 | 6313.02±2998.67 | 3369.62±0.20 | 5546.42±2218.36 | 3369.41±0.25 |
| Com_119 | Ethanolamine | 16.60 | 117459.58±8881.17 | 92523.57±26127.49 | 132746.89±54101.37 | 27123.66±2767.97 | 58338.32±2045.78 | 24193.13±3102.81 |
| Com_84 | 2-Deoxy-D-galactose 2 | 16.65 | 11715.85±4790.57 | 12740.43±3181.40 | 74319.63±18641.47 | 11900.50±1615.49 | 6204.26±2035.27 | 18137.46±1353.06 |
| Com_198 | glucose-6-phosphate 1 | 16.70 | 7254.72±0.26 | 48445.82±24209.48 | 7255.07±0.07 | 9672.72±2463.51 | 34088.86±1930.40 | 19889.50±6461.28 |
| Com_23 | benzoic acid | 16.79 | 1831880.17±153457.56 | 4144194.32±69472.18 | 4816224.38±728142.28 | 3300891.37±58163.98 | 2081441.90±228667.79 | 5541754.31±231614.04 |
| Com_78 | Guanidinosuccinic acid 4 | 16.80 | 111649.77±29030.19 | 123190.59±1925.66 | 318670.93±40138.95 | 176383.53±7169.32 | 159609.91±19697.71 | 243863.90±8624.50 |
| Com_199 | N-Methyl-L-glutamic acid 1 | 16.87 | 15321.78±2035.43 | 18230.16±5105.05 | 277599.77±107719.05 | 19047.62±2502.01 | 28866.61±962.51 | 19646.75±3058.12 |
| Com_142 | 4-Methylbenzyl alcohol | 17.05 | 50314.31±2377.31 | 40769.55±7261.39 | 20280.55±8355.24 | 45768.41±1228.77 | 42507.01±2314.94 | 43338.64±894.86 |
| Com_91 | threonine 1 | 17.08 | 63668.05±1256.39 | 65135.16±17477.57 | 292656.46±39083.67 | 40142.25±7591.29 | 27546.50±2592.94 | 56860.67±528.56 |
| Com_217 | ribitol | 17.10 | 46745.55±0.30 | 46745.74±0.11 | 242934.19±199886.95 | 200809.03±10347.24 | 117117.62±13851.61 | 151867.34±19671.74 |
| Com_42 | p-cresol | 17.16 | 1153633.90±40567.82 | 653743.67±102959.55 | 2097931.63±490169.98 | 315713.81±2101.32 | 707124.53±35423.47 | 642436.93±10049.22 |
| Com_10 | 5-aminovaleric acid lactam | 17.30 | 16996.79±15704.97 | 26260.69±25143.58 | 1582.75±0.07 | 40663.29±20669.36 | 44637.77±22358.96 | 49356.64±24374.81 |
| Com_188 | L-Allothreonine 1 | 17.31 | 1013.77±653.91 | 1153.96±402.19 | 32547.99±2998.89 | 908.62±278.78 | 4325.82±2292.77 | 49724.17±25148.85 |
| Com_144 | 3,6-Anhydro-D-galactose 3 | 17.42 | 88553.85±36120.23 | 117092.52±32687.31 | 53102.04±0.11 | 53102.15±0.22 | 53101.85±0.25 | 53101.91±0.25 |
| Com_53 | raffinose | 17.49 | 642329.24±311633.00 | 2422910.41±143092.06 | 784785.20±28331.13 | 76258.32±8031.69 | 451746.46±34159.55 | 227151.62±102242.27 |
| Com_169 | trehalose-6-phosphate | 17.57 | 22741.03±11326.10 | 15714.44±7534.36 | 4627.28±4187.25 | 14151.89±6969.82 | 11936.47±5822.03 | 23044.35±11533.88 |
| Com_93 | Fructose 2,6-biphosphate degr prod 2 | 17.80 | 3808055.50±44467.26 | 3333132.60±38535.59 | 5108816.01±2429224.19 | 3441308.48±45853.60 | 3221030.25±19465.26 | 3014851.29±687966.40 |
| Com_154 | 4-hydroxycinnamic acid | 17.84 | 109890.43±28409.83 | 127024.63±6246.38 | 54775.76±0.30 | 177582.59±62736.81 | 208242.35±19253.82 | 98010.61±22808.65 |
| Com_205 | 1,3-diaminopropane | 17.88 | 103548.14±6766.99 | 104121.49±39669.04 | 2037534.63±506193.13 | 115184.04±22428.20 | 287789.11±32102.19 | 116240.02±9935.37 |
| Com_175 | 3-Hydroxypyridine | 17.98 | 1568251.14±1530262.15 | 2577642.50±1288089.05 | 292771.47±161286.14 | 110679.86±22605.71 | 4116222.54±177061.78 | 609155.27±297657.01 |
| Com_24 | N-Acetyl-D-galactosamine 1 | 18.06 | 844939.58±65445.48 | 1206111.96±351866.44 | 8643299.40±1449315.03 | 348811.93±23695.87 | 992154.83±64381.36 | 599069.89±49082.50 |
| Com_18 | lauric acid | 18.11 | 16692.73±6432.95 | 49121.12±1705.59 | 10776.61±6492.54 | 9451.08±2594.58 | 46667.36±1273.39 | 11387.25±1383.43 |
| Com_14 | 2-Amino-1-phenylethanol | 18.16 | 14805.82±5187.41 | 4825.54±0.30 | 10680.92±5965.69 | 11116.98±3416.91 | 23972.72±1118.25 | 11234.05±3299.69 |
| Com_189 | 2,3-Dihydroxypyridine | 18.22 | 6747.43±0.26 | 6747.20±0.14 | 9952.92±3265.62 | 6747.50±0.20 | 6747.26±0.27 | 13656.06±4112.67 |
| Com_48 | L-Malic acid | 18.23 | 700105.93±76357.34 | 323030.96±32374.36 | 1465694.07±382462.12 | 383872.00±75739.97 | 838102.72±75819.22 | 361800.80±56707.70 |
| Com_128 | trehalose | 18.31 | 143711.53±3871.26 | 91141.65±28558.52 | 122640.00±36864.38 | 101375.37±17288.23 | 132034.37±4026.17 | 151807.59±2328.51 |
| Com_151 | linolenic acid | 18.32 | 145011.68±10065.16 | 69992.93±22053.29 | 130776.47±49142.24 | 81298.51±15232.22 | 129623.16±3182.35 | 97800.05±9277.28 |
| Com_172 | N-Methyl-DL-alanine | 18.55 | 58550.75±12411.02 | 109065.85±56009.82 | 24178.70±10557.35 | 25333.20±1253.75 | 81062.10±11462.65 | 65943.25±10172.35 |
| Com_105 | Cholesterol-2,2,3,4,4,6-d6 | 18.59 | 248618.51±29911.57 | 825858.21±323254.99 | 201952.70±89631.94 | 2664.60±982.56 | 155148.26±24813.38 | 54582.08±3611.75 |
| Com_153 | Citraconic acid degr1 | 18.92 | 98509.41±7774.03 | 29035.83±12083.32 | 30682.99±13242.25 | 26378.00±1237.39 | 41371.85±4133.41 | 60127.76±9358.00 |
| Com_121 | terephthalic acid | 18.99 | 226449.94±53113.86 | 305501.04±129895.03 | 102030.54±32143.32 | 86334.75±4896.68 | 229748.25±43298.38 | 190902.31±57887.88 |
| Com_117 | glycine 2 | 19.14 | 18266339.49±7447152.82 | 15685411.00±900939.17 | 13498370.47±1384000.97 | 13795764.00±80741.48 | 17863443.87±299017.07 | 17049523.07±633857.41 |
| Com_54 | N-Ethylglycine 1 | 19.26 | 74767.77±11520.62 | 54143.78±13572.03 | 55142.65±27278.48 | 77443.94±2979.70 | 65336.46±1221.95 | 83805.75±19788.69 |
| Com_174 | Gluconic lactone 1 | 19.28 | 27850.41±5022.68 | 13010.85±6149.58 | 9643.13±4979.31 | 4756.00±0.11 | 4756.11±0.22 | 21977.35±15109.09 |
| Com_166 | Melezitose | 19.36 | 408073.56±83628.30 | 299965.35±61223.04 | 94049.38±48520.08 | 114925.26±4355.64 | 78102.51±2541.07 | 180275.98±44530.47 |
| Com_176 | isocitric acid 2 | 19.42 | 104086.64±28150.97 | 66712.28±21503.59 | 6259.71±1594.27 | 50049.40±3176.99 | 78452.46±11188.50 | 86028.11±32083.91 |
| Com_108 | Galactinol 1 | 19.47 | 4820051.62±343504.05 | 3181210.99±712707.05 | 2413025.84±1591757.10 | 2482509.01±83043.14 | 3082363.34±119251.54 | 4699207.52±247620.95 |
| Com_145 | 2-ketobutyric acid 2 | 19.55 | 39326.60±7237.92 | 40544.77±5224.56 | 13821.51±8259.93 | 9694.10±373.26 | 21050.00±1380.56 | 17830.77±4013.51 |
| Com_158 | pentadecanoic acid | 19.60 | 86010.86±19686.41 | 22339.28±2295.60 | 143137.72±123364.73 | 16328.13±1089.98 | 39653.27±3157.04 | 37336.10±2449.44 |
| Com_173 | Tricetin | 19.65 | 112658.32±24139.69 | 55087.73±8438.15 | 27959.36±21222.77 | 50824.60±488.10 | 28522.78±643.28 | 72753.76±6566.53 |
| Com_159 | L-Threose 2 | 19.77 | 52176.72±12450.14 | 21986.65±1870.29 | 22239.54±5230.08 | 26060.99±1627.53 | 17061.88±2452.43 | 36112.96±3828.83 |
| Com_196 | aspartic acid 2 | 19.83 | 32612.48±7217.26 | 4259.29±558.01 | 213207.31±96308.76 | 1690.23±430.36 | 1267.94±0.26 | 13325.26±1189.35 |
| Com_57 | 3-hydroxybutyric acid | 19.94 | 14362.10±2300.68 | 5314.58±1909.81 | 15150.55±8391.01 | 12458.08±856.64 | 17687.96±783.82 | 11450.35±1240.33 |
| Com_73 | Pyruvic acid | 20.23 | 87000.87±15803.76 | 109033.22±9792.66 | 96220.99±42960.66 | 86750.07±5411.24 | 76541.99±8047.83 | 71506.52±4223.47 |
| Com_44 | fructose 2 | 20.56 | 53703.49±2488.93 | 49266.91±20332.94 | 49560.94±25760.71 | 27706.18±2810.01 | 27892.76±2666.75 | 45539.32±1623.32 |
| Com_28 | 5-Methoxytryptamine 2 | 20.61 | 202587.51±739.03 | 181590.01±78844.36 | 105271.42±41768.24 | 227899.24±2972.44 | 177699.43±9736.75 | 242915.91±6096.70 |
| Com_11 | 1-Indanol | 20.68 | 638464.05±288504.86 | 1336946.07±636868.52 | 1478252.54±214197.07 | 1379452.91±19340.74 | 1878371.18±81784.50 | 928511.47±523124.21 |
| Com_115 | Methyl jasmonate 4 | 20.72 | 2162750.09±299865.85 | 1455343.95±40110.33 | 1377713.78±327972.30 | 1190080.66±33183.75 | 1590924.65±23691.95 | 1457730.23±26100.33 |
| Com_155 | Methyl Palmitoleate | 20.73 | 25347.66±308.91 | 43830.26±2477.83 | 30774.08±9947.27 | 38699.18±2948.65 | 36689.09±15029.81 | 37236.67±2270.99 |
| Com_96 | malonic acid 1 | 20.90 | 22603.48±6371.47 | 13513.92±3442.08 | 10136.05±0.13 | 10135.93±0.20 | 21205.61±6016.19 | 14078.54±4017.05 |
| Com_140 | α-Santonin 2 | 20.92 | 10122419.53±374504.35 | 6936036.68±719933.86 | 3278478.65±1595924.25 | 6248297.53±314568.47 | 7304111.49±296490.65 | 6257485.81±702516.89 |
| Com_186 | Cerotinic acid | 21.20 | 4162.21±2165.33 | 7890.89±342.21 | 97.38±0.14 | 1618.35±1549.11 | 97.68±0.20 | 1654.21±1586.39 |
| Com_31 | sorbose 1 | 21.25 | 2722.51±0.26 | 45077.39±5113.32 | 5659.85±1794.99 | 2722.50±0.11 | 2722.61±0.22 | 2722.32±0.25 |
| Com_161 | fructose-6-phosphate | 21.27 | 149815.01±30787.20 | 237024.85±33950.01 | 79024.80±51764.56 | 282.94±148.12 | 8377.19±1906.48 | 3311.36±1861.88 |
| Com_179 | sorbitol | 21.55 | 72004.69±11460.96 | 26820.81±10200.22 | 10247.96±0.26 | 10248.23±0.13 | 70569.42±5166.63 | 10248.11±0.20 |
| Com_148 | 2-Amino-2-norbornanecarboxylic acid 3 | 21.94 | 1077723.69±39181.92 | 1003720.83±6470.84 | 238950.12±17003.57 | 349004.29±2812.08 | 733169.11±30923.08 | 1041512.11±30041.05 |
| Com_56 | hydroxylamine | 22.27 | 48898.19±3581.40 | 46470.68±8043.87 | 19770.74±5316.63 | 67287.86±2182.26 | 28639.67±3528.01 | 45919.17±2994.15 |
| Com_178 | valine | 22.31 | 494906.18±6171.86 | 242302.66±14818.38 | 15747.72±8203.77 | 8993.53±2874.86 | 282883.62±9879.33 | 13432.26±687.19 |
| Com_163 | conduritol b epoxide 2 | 22.34 | 111080.27±3647.77 | 51422.30±1422.70 | 22986.74±7394.07 | 22902.13±1079.91 | 55925.94±4926.27 | 18037.01±1876.17 |
| Com_201 | 2-Butyne-1,4-diol | 22.49 | 46911.64±2907.89 | 34809.42±3778.29 | 93540.26±43916.41 | 44154.76±1981.24 | 53353.00±3460.63 | 43307.59±3194.02 |
| Com_177 | oxalacetic acid | 22.55 | 29218.87±2876.29 | 15897.88±2013.58 | 4526.37±0.26 | 4526.64±0.13 | 15148.83±788.83 | 4526.53±0.20 |
| Com_162 | phenylalanine 1 | 22.56 | 201510.29±4006.61 | 49672.05±1628.78 | 68108.25±40172.50 | 61738.45±743.67 | 64938.70±1767.49 | 45772.16±449.34 |
| Com_192 | tyrosine 1 | 22.69 | 50794.65±7017.28 | 23138.94±4377.88 | 241164.29±59469.64 | 312434.47±44873.66 | 1422269.76±112758.92 | 51293.07±5938.64 |
| Com_160 | linoleic acid | 22.79 | 100275.01±3126.49 | 50468.11±2148.38 | 5799.41±0.26 | 5799.18±0.14 | 38300.00±1288.63 | 5799.68±0.07 |
| Com_137 | xylose 1 | 22.85 | 168831.48±4990.44 | 139819.11±7936.63 | 115878.66±38054.88 | 100056.06±1814.15 | 154262.77±13574.86 | 147887.99±18857.16 |
| Com_111 | Threonic acid | 22.97 | 417885.77±5527.76 | 336324.03±36045.04 | 349158.93±99624.72 | 195222.63±37338.36 | 216857.03±19967.30 | 301103.02±34181.94 |
| Com_104 | Dioctyl phthalate | 23.27 | 132.09±0.30 | 5150.50±2705.91 | 132.46±0.13 | 175.99±44.59 | 132.08±0.25 | 342.52±214.21 |
| Com_156 | 9-Fluorenone 2 | 23.42 | 29906.68±1929.10 | 15389.86±7135.46 | 18591.57±10397.83 | 18321.93±969.95 | 21922.22±6917.82 | 8386.75±0.07 |
| Com_157 | Pipecolinic acid | 23.43 | 170512.57±5854.16 | 95043.88±22435.11 | 125989.97±38070.88 | 99795.82±2523.59 | 84931.19±8894.19 | 75822.69±9313.74 |
| Com_168 | Zymosterol 2 | 23.50 | 74038.56±9555.32 | 24277.24±9082.79 | 10602.86±0.14 | 10603.36±0.07 | 30715.61±4906.58 | 10602.94±0.23 |
| Com_131 | Isoleucine | 23.51 | 35331.21±691.44 | 16112.46±2498.03 | 9465.61±2963.71 | 4546.22±0.30 | 14663.53±1222.46 | 4546.40±0.11 |
| Com_123 | kyotorphin 2 | 23.54 | 19998.98±1383.72 | 7874.26±3746.83 | 3166.08±2943.85 | 276.75±0.11 | 8534.52±766.25 | 896.88±631.50 |
| Com_110 | 4-Pyridoxic acid | 23.69 | 246927.35±27178.47 | 113986.21±22898.93 | 163850.30±24884.78 | 71612.94±788.99 | 78543.02±10160.43 | 99848.50±10813.70 |
| Com_167 | 6-phosphogluconic acid | 23.99 | 17861.36±1496.54 | 11806.73±2954.61 | 6377.40±0.30 | 6377.59±0.11 | 6377.70±0.22 | 6377.40±0.25 |
| Com_103 | alanine 1 | 24.35 | 21778.43±0.26 | 76728.84±7605.21 | 46355.60±12960.17 | 21778.79±0.07 | 21778.37±0.23 | 21778.37±0.26 |
| Com_191 | glycerol | 24.46 | 7074.70±0.30 | 41512.90±5911.13 | 27154.64±7999.56 | 41779.33±9304.70 | 29742.70±726.81 | 30255.00±1429.89 |
| Com_219 | salicylic acid | 24.47 | 18193.16±0.26 | 63943.62±46612.85 | 76908.13±4288.79 | 93790.65±29599.92 | 18193.35±0.22 | 18193.05±0.25 |
| Com_182 | Citraconic acid 1 | 24.71 | 695115.34±31440.29 | 92945.17±71628.72 | 600368.35±40653.85 | 511799.83±71866.95 | 668571.73±12369.26 | 346356.88±32440.41 |
| Com_134 | naringin | 24.76 | 26491.69±1795.65 | 6500.78±5510.41 | 669736.36±42308.14 | 8173.51±4101.64 | 10230.73±1147.10 | 30832.57±5492.83 |
| Com_40 | sulfuric acid | 24.82 | 1211607.51±40633.46 | 206899.49±24973.82 | 2747947.88±142872.21 | 746087.23±86270.24 | 1313428.27±23497.84 | 453105.63±146524.39 |
| Com_129 | 2,4-diaminobutyric acid 3 | 24.87 | 5840.62±2708.30 | 7863.06±4769.17 | 10979.90±7944.36 | 10926.58±3997.81 | 6046.39±1599.34 | 3182.32±0.23 |
| Com_132 | Phenylacetamide | 24.96 | 11884.52±3026.91 | 8913.66±0.11 | 8913.77±0.22 | 8913.48±0.25 | 8913.53±0.25 | 8913.61±0.09 |
| Com_181 | Sucrose-6-Phosphate | 25.14 | 80413.63±4579.02 | 25772.76±12215.77 | 481230.36±41521.71 | 2229.79±0.30 | 2972.89±757.02 | 35648.58±2226.34 |
| Com_43 | fructose 1 | 25.32 | 76310.61±6773.35 | 19999.85±17766.44 | 66626.61±11697.89 | 3416.13±869.89 | 25215.23±11987.88 | 73555.65±6581.96 |
| Com_184 | Monoolein | 25.56 | 42169.10±3074.47 | 8865.87±4104.61 | 29230.07±4211.53 | 10497.78±4522.55 | 73255.20±6875.03 | 45431.70±3709.29 |
| Com_171 | 6-deoxy-D-glucose 2 | 25.64 | 761225.80±76678.01 | 427992.55±221552.89 | 580379.51±70235.33 | 1708836.71±867248.41 | 1267080.82±152038.82 | 1683549.34±251894.55 |
| Com_164 | citric acid | 25.87 | 5601.39±2076.29 | 2050.08±0.14 | 2050.59±0.07 | 2050.17±0.23 | 4375.68±1268.76 | 2733.41±695.98 |
| Com_187 | 2-mercaptoethanesulfonic acid 2 | 26.44 | 137402.39±27873.08 | 92800.76±16001.70 | 93430.69±12853.73 | 47892.87±14219.18 | 50457.67±24745.93 | 234601.52±101108.89 |
| Com_170 | 4-aminobutyric acid 1 | 26.62 | 19993.06±168.09 | 11904.63±1600.79 | 4114.88±2025.84 | 1456.64±1337.61 | 12038.67±6090.34 | 1581.18±732.67 |
| Com_185 | Pelargonic acid | 26.84 | 538166.84±89746.25 | 226870.94±28821.90 | 134013.43±19179.29 | 68955.32±26293.74 | 221571.95±114581.39 | 223657.54±35340.14 |
| Com_141 | 4-Acetylbutyric acid 2 | 27.94 | 41779.46±4265.64 | 10686.80±5743.24 | 18961.25±3132.45 | 18375.05±304.30 | 22744.18±11610.19 | 36586.20±14540.07 |
| Com_100 | myo-inositol | 28.02 | 34606.68±0.26 | 34606.45±0.14 | 34606.95±0.07 | 34606.53±0.23 | 34606.53±0.26 | 34606.47±0.12 |
| Com_120 | N-ethylmaleamic acid 3 | 28.48 | 52580.51±34509.93 | 40677.76±32573.21 | 61358.34±8724.53 | 43708.15±19458.97 | 25820.84±11953.90 | 63722.20±47678.91 |
| Com_147 | 3-Cyanoalanine | 28.68 | 76434.12±33837.02 | 37079.60±20833.68 | 45969.77±9643.69 | 33101.58±10949.71 | 36558.88±5996.21 | 30850.39±11027.71 |
| Com_193 | Arachidic acid | 29.15 | 1926.30±490.41 | 1444.77±0.14 | 63535.02±22074.58 | 1445.27±0.07 | 1444.85±0.23 | 1444.85±0.26 |
| Com_83 | succinic acid | 29.22 | 100526.07±55104.44 | 92936.83±30660.76 | 89054.98±13756.03 | 69312.89±37512.31 | 127303.20±19066.63 | 93204.02±49894.99 |
| Com_190 | 24,25-dihydrolanosterol | 29.23 | 439.78±0.26 | 440.05±0.13 | 439.94±0.20 | 439.70±0.27 | 2223.86±1599.49 | 763.39±329.82 |
| Com_139 | Phytol | 29.55 | 577541.44±156185.39 | 486748.44±174092.35 | 1000818.06±22933.28 | 612136.92±262951.64 | 478867.80±153885.80 | 794125.34±176358.51 |
| Com_118 | 1,4-Cyclohexanedione 1 | 30.35 | 244125.31±113673.04 | 443523.85±167132.43 | 635473.17±63476.87 | 18521.39±7344.28 | 95888.87±37576.00 | 114481.34±56315.94 |
| Com_122 | d-Glucoheptose 1 | 30.84 | 136608.42±70814.09 | 500492.18±110184.61 | 5165295.46±2051326.23 | 7434.19±1893.43 | 30605.25±19070.76 | 234385.65±48001.85 |

**Table S1 All metabolites identified based on GC-MS in rice of all kinds.**

CH, HM, NX, YX, HY and MX stand for Changhui 871, Huaxiang Madi, Nongxiang 39, Yahe xiang, Huaxiang Yousi, and Meixiangzhan 2, respectively; all data was present as mean ± SE (n=3).
