# Supplementary material for: Non-targeted GC–MS metabolomics-based differences in Indica rice seeds of different varieties
Source: BMC Plant Biol. 2024 Jun 8;24:519. doi: 10.1186/s12870-024-05255-6 (PMC11162567; doi:10.1186/s12870-024-05255-6)
Supplement: Supplementary file 4 — Supplementary Material 4. [file 12870_2024_5255_MOESM4_ESM.docx]

| ID | metabolite | RT (min) | CH | HM | NX | YX | HY | MX |
| --- | --- | --- | --- | --- | --- | --- | --- | --- |
| Com_1 | L-Allothreonine 1 | 6.92 | 2253464.25±177348.33 | -- | 2377985.17±156239.70 | -- | -- | -- |
| Com_4 | glycerol | 7.20 | 533024.79±17852.23 | -- | -- | 561759.22±15774.20 | -- | 532519.97±9045.94 |
| Com_5 | tyrosine 1 | 7.41 | 390458.96±31842.31 | -- | 314801.17±16861.84 | -- | -- | -- |
| Com_6 | Arachidic acid | 7.79 | 7822227.69±200282.15 | 9604623.91±697539.09 | -- | -- | 13394515.40±492180.05 | 4074587.69±150673.59 |
| Com_10 | Phenyl β-D-glucopyranoside | 8.17 | 77078.86±19962.47 | -- | -- | 75691.73±6662.44 | 84048.40±14452.82 | -- |
| Com_11 | glucose-6-phosphate 1 | 8.33 | 8665420.43±1253234.13 | -- | 8544552.41±1290032.86 | 9097278.72±1607510.92 | -- | 9128196.27±1350645.33 |
| Com_12 | N-Methyl-L-glutamic acid 1 | 8.35 | 95924.32±42329.34 | 76594.50±30712.30 | -- | -- | 61968.55±44860.97 | 98902.26±41310.15 |
| Com_21 | Dehydroepiandrosterone | 8.84 | 709425.85±87525.89 | 760172.16±90525.61 | -- | -- | -- | -- |
| Com_27 | Myristic Acid | 9.57 | 38154.86±2243.75 | -- | -- | 33875.60±6569.94 | -- | -- |
| Com_29 | Gentiobiose 1 | 9.80 | 64250.53±2901.60 | -- | -- | 83876.03±3701.47 | -- | -- |
| Com_35 | oxoproline | 10.08 | 907638.91±171100.67 | -- | 1625606.20±205783.67 | -- | -- | -- |
| Com_36 | Abietic Acid 1 | 10.15 | 7348732.61±311891.45 | -- | 6016403.62±554165.59 | 3761616.81±193489.25 | 6147854.03±298176.84 | 5822955.41±181684.04 |
| Com_38 | 1,5-Anhydroglucitol | 10.18 | 14654.36±9097.79 | -- | 190810.29±58599.10 | -- | -- | -- |
| Com_39 | Allantoic acid 3 | 10.28 | 343520.24±18609.66 | -- | 348848.77±21719.01 | -- | -- | -- |
| Com_41 | allose 1 | 10.39 | 39580.07±1563.58 | -- | -- | 11062.32±0.26 | 24466.52±1246.25 | -- |
| Com_43 | β-Alanine 1 | 10.53 | 2001342.94±54162.18 | -- | -- | 439711.67±39442.03 | -- | -- |
| Com_44 | 5-aminovaleric acid lactam | 10.57 | 39951.72±5261.82 | -- | -- | 21065.44±4746.42 | -- | -- |
| Com_46 | D-Glyceric acid | 10.69 | 22206.40±2574.97 | -- | 18493.54±1352.23 | 24163.62±3089.32 | -- | -- |
| Com_48 | 2-Amino-1-phenylethanol | 10.75 | 402970.53±64580.27 | -- | 413116.51±63640.10 | -- | 556244.89±40746.67 | -- |
| Com_51 | N-Acetyl-β-D-mannosamine 4 | 10.92 | 157665.80±1497.64 | -- | -- | -- | 173812.65±14323.41 | -- |
| Com_52 | lauric acid | 10.93 | 119603.04±18074.54 | -- | -- | -- | -- | 91000.56±6323.61 |
| Com_55 | lactulose 1 | 11.24 | 27762.37±955.85 | 130251.20±31005.23 | -- | 23335.79±1658.27 | 52316.22±9092.97 | 135284.02±4440.94 |
| Com_56 | Threitol | 11.34 | 21947.79±8031.25 | 6188.50±0.14 | -- | 21744.70±8275.39 | -- | -- |
| Com_58 | N-Acetyl-D-galactosamine 1 | 11.40 | 47425.27±5608.75 | -- | -- | -- | 40689.94±9083.73 | -- |
| Com_59 | L-cysteine | 11.42 | 164699.43±14697.65 | -- | 34352.45±12548.85 | -- | -- | -- |
| Com_61 | fumaric acid | 11.54 | 59771.11±8853.38 | 44231.98±12691.62 | -- | -- | -- | 54723.25±6982.56 |
| Com_62 | 5-Methoxytryptamine 2 | 11.58 | 12818.23±0.26 | 12818.00±0.14 | 12818.51±0.07 | 12818.08±0.23 | 12818.09±0.26 | -- |
| Com_64 | maltotriose 1 | 11.69 | 73611.38±7442.60 | -- | 184424.10±83124.41 | 50238.31±4067.25 | -- | -- |
| Com_67 | epsilon-Caprolactam | 11.93 | 10553.45±2636.41 | -- | 6132.83±669.10 | -- | 8763.28±3485.68 | -- |
| Com_68 | uridine 2 | 11.96 | 34620.95±2539.99 | -- | 45551.78±14277.16 | 59879.83±4067.92 | -- | 32657.23±5392.27 |
| Com_71 | xylitol | 12.50 | 9785.10±0.30 | -- | -- | 9785.36±0.20 | -- | 18047.24±4480.12 |
| Com_72 | sucrose | 12.50 | 67496.85±10896.03 | -- | -- | -- | 79946.08±5134.58 | 75719.96±3617.01 |
| Com_73 | 2-hydroxybutanoic acid | 12.51 | 14613.33±1692.35 | -- | -- | -- | 11743.61±4060.39 | -- |
| Com_77 | fructose 1 | 12.69 | 35569.48±2870.03 | -- | 40320.77±10021.72 | -- | -- | -- |
| Com_79 | 2-hydroxy-3-isopropylbutanedioic acid | 12.82 | 7902.50±1794.65 | 10540.73±3819.16 | -- | 6609.24±2180.89 | 14460.46±5093.09 | -- |
| Com_80 | lysine | 12.93 | 20064.27±8623.46 | -- | 14518.30±4352.13 | -- | 23389.69±1231.32 | -- |
| Com_81 | 2-Monopalmitin | 13.00 | 457921.64±10644.20 | -- | -- | -- | -- | 1504524.83±49974.69 |
| Com_82 | 2-hydroxy-3-isopropylbutanedioic acid | 13.13 | 39604.10±1164.79 | 94097.63±4716.66 | -- | -- | 40340.99±3378.83 | 50601.65±3134.95 |
| Com_83 | Dehydroabietic Acid | 13.22 | 1420347.71±22601.52 | -- | 5710114.55±1579235.81 | 1015925.26±52685.64 | -- | 825880.34±11368.29 |
| Com_84 | gluconic acid 1 | 13.29 | 28298.10±0.30 | -- | -- | 28298.47±0.13 | -- | 28298.12±0.27 |
| Com_85 | Itaconic acid | 13.30 | 64584.51±3308.92 | -- | 27561.99±5413.31 | -- | 58022.69±1124.55 | -- |
| Com_86 | uracil | 13.41 | 2215059.39±51219.28 | -- | -- | -- | -- | 2363948.94±31875.59 |
| Com_92 | citrulline 1 | 14.02 | 44363.27±2730.77 | -- | 36866.39±7341.14 | -- | -- | -- |
| Com_96 | Digitoxose 2 | 14.59 | 10533.09±0.30 | -- | 10533.38±0.22 | -- | -- | -- |
| Com_102 | maltose | 14.97 | 52889.27±599.33 | -- | 77002.19±1892.59 | -- | 32029.54±2155.73 | -- |
| Com_103 | lactose 1 | 15.02 | 6810.14±1038.71 | -- | 9119.84±343.54 | -- | -- | -- |
| Com_104 | Linoleic acid methyl ester | 15.05 | 258750.01±5291.91 | 217537.52±11041.25 | -- | 110740.78±3830.45 | -- | -- |
| Com_109 | N-Acetyl-L-leucine 1 | 15.54 | 685210.59±8959.40 | 1155468.38±36258.81 | -- | -- | -- | -- |
| Com_111 | cis-gondoic acid | 15.70 | 57639.47±53724.76 | -- | 344802.02±182603.32 | 65430.28±61662.34 | -- | 118892.72±8865.79 |
| Com_112 | Guanidinosuccinic acid 4 | 15.76 | 50692.67±21586.07 | -- | -- | 63483.83±1910.41 | 65145.03±586.99 | -- |
| Com_118 | 2-Deoxy-D-galactose 2 | 16.19 | 3777043.70±19587.50 | -- | 5216096.14±291639.13 | 2260800.50±59753.69 | -- | -- |
| Com_119 | androsterone 1 | 16.28 | 11275.85±9601.60 | 11879.02±11518.92 | -- | 26655.71±1959.72 | -- | 26744.15±13531.83 |
| Com_122 | asparagine 1 | 16.59 | 3369.36±0.30 | -- | 6313.02±2998.67 | -- | 5546.42±2218.36 | -- |
| Com_123 | asparagine 4 | 16.60 | 117459.58±8881.17 | -- | -- | -- | 58338.32±2045.78 | -- |
| Com_129 | 2-Deoxyerythritol | 17.05 | 50314.31±2377.31 | -- | -- | 45768.41±1228.77 | -- | -- |
| Com_132 | glutamic acid | 17.16 | 1153633.90±40567.82 | -- | 2097931.63±490169.98 | -- | -- | 642436.93±10049.22 |
| Com_133 | azelaic acid | 17.30 | 16996.79±15704.97 | -- | -- | 40663.29±20669.36 | -- | 49356.64±24374.81 |
| Com_135 | N-α-Acetyl-L-ornithine 3 | 17.42 | 88553.85±36120.23 | -- | 53102.04±0.11 | -- | -- | -- |
| Com_136 | Sophorose 2 | 17.49 | 642329.24±311633.00 | -- | 784785.20±28331.13 | 76258.32±8031.69 | 451746.46±34159.55 | -- |
| Com_137 | alanine 1 | 17.57 | 22741.03±11326.10 | -- | -- | 14151.89±6969.82 | -- | -- |
| Com_140 | Tagatose 1 | 17.88 | 103548.14±6766.99 | -- | -- | 115184.04±22428.20 | 287789.11±32102.19 | 116240.02±9935.37 |
| Com_145 | Threonic acid | 18.22 | 6747.43±0.26 | -- | 9952.92±3265.62 | -- | -- | -- |
| Com_146 | proline | 18.23 | 700105.93±76357.34 | -- | 1465694.07±382462.12 | 383872.00±75739.97 | -- | -- |
| Com_150 | glycine 1 | 18.59 | 248618.51±29911.57 | -- | -- | 2664.60±982.56 | -- | -- |
| Com_151 | glycine 2 | 18.92 | 98509.41±7774.03 | -- | 30682.99±13242.25 | 26378.00±1237.39 | -- | -- |
| Com_154 | N-ethylmaleamic acid 3 | 19.26 | 74767.77±11520.62 | -- | -- | -- | -- | 83805.75±19788.69 |
| Com_156 | d-Glucoheptose 1 | 19.36 | 408073.56±83628.30 | -- | -- | -- | 78102.51±2541.07 | -- |
| Com_157 | kyotorphin 2 | 19.42 | 104086.64±28150.97 | -- | -- | 50049.40±3176.99 | -- | 86028.11±32083.91 |
| Com_159 | Glucoheptonic acid 3 | 19.55 | 39326.60±7237.92 | 40544.77±5224.56 | 13821.51±8259.93 | 9694.10±373.26 | 21050.00±1380.56 | -- |
| Com_162 | trehalose | 19.77 | 52176.72±12450.14 | 21986.65±1870.29 | -- | -- | -- | -- |
| Com_164 | 1-Monopalmitin | 19.94 | 14362.10±2300.68 | -- | -- | -- | 17687.96±783.82 | -- |
| Com_165 | Isoleucine | 20.23 | 87000.87±15803.76 | -- | 96220.99±42960.66 | -- | -- | -- |
| Com_168 | naringin | 20.68 | 638464.05±288504.86 | -- | 1478252.54±214197.07 | -- | -- | -- |
| Com_169 | Ribonic acid, γ-lactone | 20.72 | 2162750.09±299865.85 | -- | -- | 1190080.66±33183.75 | -- | 1457730.23±26100.33 |
| Com_170 | Aminomalonic acid | 20.73 | 25347.66±308.91 | -- | -- | 38699.18±2948.65 | -- | -- |
| Com_172 | shikimic acid | 20.92 | 10122419.53±374504.35 | -- | -- | -- | 7304111.49±296490.65 | -- |
| Com_177 | phytosphingosine 1 | 21.94 | 1077723.69±39181.92 | -- | 238950.12±17003.57 | 349004.29±2812.08 | -- | 1041512.11±30041.05 |
| Com_178 | 3,6-Anhydro-D-galactose 3 | 22.27 | 48898.19±3581.40 | 46470.68±8043.87 | -- | 67287.86±2182.26 | 28639.67±3528.01 | 45919.17±2994.15 |
| Com_180 | serine 1 | 22.34 | 111080.27±3647.77 | -- | 22986.74±7394.07 | -- | -- | -- |
| Com_183 | maleic acid | 22.56 | 201510.29±4006.61 | -- | -- | -- | 64938.70±1767.49 | 45772.16±449.34 |
| Com_191 | Pipecolinic acid | 23.50 | 74038.56±9555.32 | -- | 10602.86±0.14 | -- | 30715.61±4906.58 | -- |
| Com_195 | fructose-6-phosphate | 23.99 | 17861.36±1496.54 | 11806.73±2954.61 | -- | -- | -- | -- |
| Com_196 | phenylalanine 1 | 24.35 | 21778.43±0.26 | -- | 46355.60±12960.17 | 21778.79±0.07 | -- | -- |
| Com_198 | citric acid | 24.47 | 18193.16±0.26 | 63943.62±46612.85 | -- | -- | -- | 18193.05±0.25 |
| Com_199 | palmitoleic acid | 24.71 | 695115.34±31440.29 | -- | -- | 511799.83±71866.95 | -- | -- |
| Com_200 | Melezitose | 24.76 | 26491.69±1795.65 | -- | -- | 8173.51±4101.64 | -- | -- |
| Com_204 | 4-aminobutyric acid 1 | 25.14 | 80413.63±4579.02 | -- | -- | 2229.79±0.30 | -- | -- |
| Com_205 | 6-deoxy-D-glucose 2 | 25.32 | 76310.61±6773.35 | -- | 66626.61±11697.89 | -- | -- | -- |
| Com_206 | N-Methyl-DL-alanine | 25.56 | 42169.10±3074.47 | -- | -- | 10497.78±4522.55 | -- | -- |
| Com_212 | valine | 27.94 | 41779.46±4265.64 | -- | 18961.25±3132.45 | -- | -- | -- |
| Com_214 | Purine riboside | 28.48 | 52580.51±34509.93 | -- | 61358.34±8724.53 | -- | -- | -- |
| Com_217 | ferulic acid | 29.22 | 100526.07±55104.44 | -- | -- | 69312.89±37512.31 | -- | -- |
| Com_220 | Cerotinic acid | 30.35 | 244125.31±113673.04 | 443523.85±167132.43 | 635473.17±63476.87 | 18521.39±7344.28 | -- | -- |

**Table S2 Discriminating metabolites in rice seeds between CH and other varieties (HM, NX, YX, HY and MX).**

Discriminating metabolites were selected based on VIP value obtained from PLS-DA above 1 and *p* value from t-text below 0.05; CH, HM, NX, YX, HY and MX stand for Changhui 871, Huangxiang yujing, Nongxiang 39, Daoxiang, Huangxiang Yousi, and Meixiangzhan 2, respectively; --, none discriminating metabolites; all data were showed in mean ± SE (n = 3).
